# Characterization of *Cyclamen* genotypes using morphological descriptors and DNA molecular markers in a multivariate analysis

**DOI:** 10.3389/fpls.2023.1100099

**Published:** 2023-01-26

**Authors:** Mihaiela Cornea-Cipcigan, Doru Pamfil, Cristian Radu Sisea, Rodica Margaoan

**Affiliations:** ^1^ Department of Horticulture and Business in Rural Development, Faculty of Horticulture, University of Agricultural Sciences and Veterinary Medicine, Cluj-Napoca, Romania; ^2^ Research Centre for Biotechnology in Agriculture Affiliated to Romanian Academy, University of Agricultural Sciences and Veterinary Medicine, Cluj-Napoca, Romania; ^3^ Laboratory of Cell Analysis and Spectrometry, Advanced Horticultural Research Institute of Transylvania, University of Agricultural Sciences and Veterinary Medicine, Cluj-Napoca, Romania

**Keywords:** *Cyclamen*, cluster analysis, genetic diversity, morphological characterization, molecular markers

## Abstract

Morphological and molecular characterization of germplasm is essential for the improvement of cultivated plants efforts. This study investigated the genetic diversity of 32 *Cyclamen* genotypes comprising 16 C*. persicum* varieties and 16 *Cyclamen* species using multivariate analysis for 36 morphological traits (19 quantitative and 17 qualitative) and molecular characterization (SRAP and SCoT markers). The color CIELab parameters were collected *via* PCE-CSM7 that separately measured the leaves dark and silvery patterns and the flower’s slip (region of the petal top), eye (the region of the petal base) and sepal. Genetic diversity was also evaluated using Shannon Wiener (H′) and Simpson’s (λ) Indices, and Pilou evenness (J) using the library vegan from R software. According to the principal component analysis, the variables that contributed the most were leaf pattern color, leaf abaxial surface color, pedicel coiling, leaf and stem number. The color indicators of *Cyclamen* leaves showed decreased L* values in darker colored genotypes, whereas increased a* values were noticed in flower eye and lower in slip. Molecular characterization was based on 26 SRAP and 12 SCoT markers that produced clearly repeatable DNA bands and exhibited significant levels of polymorphism. Based on the morphological traits and molecular markers data, the UPGMA method for hierarchical clustering technique was used to generate the dendrograms, and their entanglement was obtained using the Tanglegram algorithm from the dendextend package with the R software. Entanglement analysis (0.30) between dendrograms obtained from the morphological and genetic analysis using SRAP markers showed a high association. Comparison between color measurements of flowers (entanglement=0.45) and leaves (entanglement=0.47) with SCoT analysis revealed differences at species level, discriminating between similar genotypes. Combined phenotypic and molecular analysis improved the comprehensive estimation of real diversity in the investigated *Cyclamen* genotypes. The findings of the present study are useful for quantifying diversity and genetic variability in *Cyclamen* breeding and genetic investigations.

## 1 Introduction

*Cyclamen* sp. are part of the Primulaceae family, being widely cultivated throughout the Mediterranean area as an ornamental plant, but also for its pharmaceutical properties. Of the 24 known species, *Cyclamen persicum* Mill. is distributed from south-central Turkey to Lebanon-Syria, being the most significant in terms of production, because of its adaptability to new ecological conditions. *C. persicum* is a much admired ornamental plant of high economic importance, especially in the Netherlands, Germany and Italy. *Cyclamen* is particularly essential in traditional and modern medicine, in addition to its decorative and commercial importance (Sarikurkcu, 2011; Turan and Mammadov, 2018; Cornea-Cipcigan et al., 2019). *Cyclamen* possesses cultural and religious relevance, symbolizing empathy and devotion in the Mediterranean culture, is cultivated in Islamic churchyards and monasteries, and is a consecrated flower in Japan (Grey-Wilson, 2015). In addition to their ornamental importance, *Cyclamen* sp. also possesses strong antioxidant properties due to the phenolic composition, and anti-cancer activities reported in HeLa, non-small cell lung cancer H1299 cells, human colorectal cancer cells (HCT 116 and HT-29), human breast adenocarcinoma (MDA-MB-231) and human fibroblasts BJ cells (Mihci-Gaidi et al., 2010a; Mihci-Gaidi et al., 2010b; Yildiz et al., 2013; Cornea-Cipcigan et al., 2022a). Due to a shortage of plant material, their use for the production of pharmaceuticals has become a severe problem, potentially leading to the loss of plant populations and variety, natural habitat deterioration, and/or species extinction. Classification of morphological traits has been done in only a few *Cyclamen* species endemic to Turkey (Curuk et al., 2015; Curuk et al., 2016).

The materials preserved in gene banks, such as wild, cultivated and selected accessions represent important sources of variability for breeders. Thus, breeders have used a variety of approaches throughout the years to investigate and assess the level of diversity in plant populations (Bhandari et al., 2017). In *Cyclamen*, genetic variation has been evaluated through morphological characters and molecular markers, such as the internal transcribed spacer regions (ITS) (Anderberg et al., 2000), random amplified polymorphic DNAs (RAPD) (Naderi et al., 2009; Taşkin et al., 2012), and sequence-related amplified polymorphisms (SRAP) (Simsek et al., 2017) (Robarts and Wolfe, 2014; Yagi et al., 2014; Samarina et al., 2021).

As the availability of specific molecular markers for *Cyclamen* is limited, SRAP markers represented the basis of the molecular investigations performed in this study. Also the PCR-based start codon targeted (SCoT) markers which were only used previously on other species were included in the analysis. Both types of markers constitute simple, reproducible and low cost techniques for characterizing germplasm collections (Collard and Mackill, 2008), with the aim of genetic diversity screening (Igwe et al., 2017), species identification or phylogenetic analysis (Jalilian et al., 2018).

Although molecular markers have been extensively used to evaluate species variety, extremely low or insignificant correlations have been observed among dissimilarity matrices generated using both phenotypic and molecular data (Gupta et al., 2018). As a result, if the non-overlapping data is derived from phenotypic and genotypic divergence matrix, combining them may offer a full picture of a population’s variety (Houmanat et al., 2021). Multivariate analysis approaches (HCA or PCA), are commonly used to precisely classify various plants based on their agro-morphological, molecular, chemical composition, or bioactivities that are regularly compared with correlation coefficients (Granato et al., 2018). To accurately evaluate the genetic variation among individuals, it is important to choose a suitable dissimilarity coefficient and hierarchical clustering method since both have an impact on the outcomes of genetic diversity analysis (Darkwa et al., 2020). Using both agronomic variables and molecular markers may contribute to a more comprehensive genetic diversity analysis within and among species, phylogenetic investigations, and fingerprinting in various plants (Jalilian et al., 2018; Long et al., 2020). Consequently, to evaluate the genetic diversity of *Cyclamen* genotypes, joint hierarchical cluster analysis was constructed between phenotypic and molecular data. Choosing the most suitable clustering method is significant to determine proper genetic dissimilarity and diversity between and within populations and clustering, since different correlations may give inconclusive or dissimilar results. To best of our knowledge, comparison between clustering methods based on phenotypic and molecular markers (SRAP and SCoT markers) has not been employed in *Cyclamen* until now. Our studies objectives were (1) the comparison of several correlation matrices and HCA for investigating genetic diversity in *Cyclamen*, and (2) the evaluation of genetic diversity and divergence in *Cyclamen* genotypes by the use of morphological, molecular, and combined data.

## 2 Materials and methods

### 2.1 Plant material

Seeds of *Cyclamen* genotypes were germinated on two-layer filter paper in Petri dishes with distilled water, as previously reported (Cornea-Cipcigan et al., 2020). After germination (~5 weeks), the seedlings were transplanted into pots and watered when necessary. The growing substrate (50/20/20/10 *v/v*) was a mixture of sowing and propagation soil (pH = 6.0) with NPK (0.1:0.01:0.03 m/m%), *Cyclamen* substrate (pH = 6.2) with NPK (1.0:0.1:0.3 m/m%), organic substances (70%) and perlite. The average greenhouse temperature was 18-22°C and 60% relative humidity. The quantitative variables were scanned and measured using the ImageJ Programme (v1.52a, Wayne Rasband, National Institutes of Health Bethesda, Maryland, USA) for image processing after plants reached maturity. Details regarding breeding companies and genotype characteristics are shown in **Supplementary Table S1**.

### 2.2 Phenotyping

**Figure 1:**
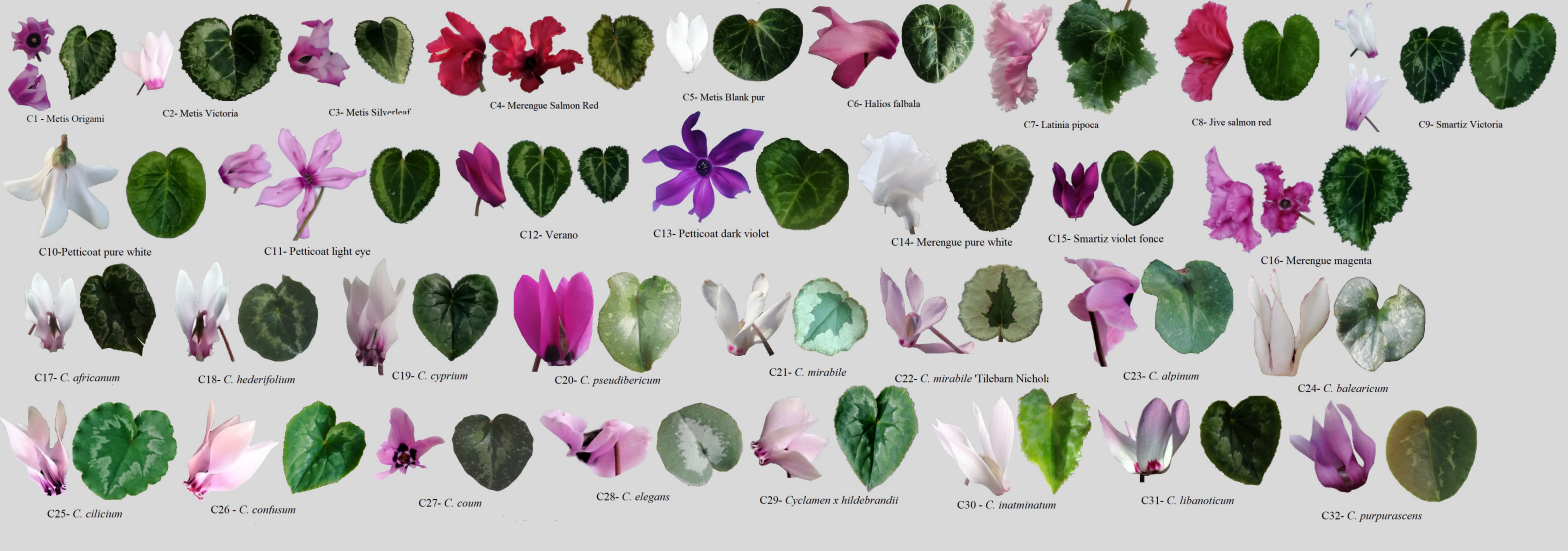
*Cyclamen* genotypes used in the study. Lane C1-C16, *C. persicum* cultivars, C17-C32, different *Cyclamen* species.

Thirty-six morpho-agronomic characters were measured for the 32 *Cyclamen* genotypes (**Figure 1**), from which 19 were quantitative and 17 qualitative. The quantitative variables assessed included number of stems/plant (NS), petiole diameter (PD), petal length (PL), petal width (PW), flower area (FL), pistil length (PIL), stamen length (SL), flower numbers (FN), pedicel length (PEDL), leaf number (LN), lamina length (LL), lamina width (LW), leaf length/width ratio (LL/W ratio), petiole length (PETL), leaf shape (LS), leaf margin (LM), leaf pattern area (LPA), leaf area (LA) and canopy area (CA) were recorded at maturity. The qualitative variables included the petiole color (PETC), plant vigor (PV), flowering intensity (FI), petal color at flowering (PCF), petal color at senescence (PCS), basal corolla ring color (BCPR), color of upper corolla (CUP), color of lower corolla (CLC), basal corolla ring diameter (BCRD), pedicel coiling (PEC), darker petal margin (DPM), stigma position (SP), lamina (degree of lobbing, LD), leaf pattern (LP), leaf patter color (LPC), leaf abaxial surface color (LASC), canopy architecture (CAR) were recorded at maturity. The quantitative and qualitative variables are shown in **Supplementary Table S2**.

### 2.3 Color measurements

To estimate the color of *Cyclamen* flowers and leaves a portable colorimeter PCE-CSM7 (PCE instruments, Meschede, Germany) was used. According to the International Commission of Illumination’s (CIE), the color spaces were expressed as *L* ^∗^, *a* ^∗^, *b* ^∗^, color intensity and shade, where L* expresses the sample’s lightness (100) to darkness (0), a* represents the greenness (negative) to redness (positive) degrees and b* the blueness (negative) to yellowness (positive) degrees. Intensity of color (*C*, chroma) and shade (*H°*, hue angle) (Clydesdale and Ahmed, 1978) were generated by the use of the equations shown below:


$$C=[{\left(a^{*}{)}^{\;2}+\;{\left(b^{*}\right)}^{2}\right]}^{0.5}$$

$$H{}^{\circ}=tan^{-1}\frac{b^{*}}{a^{*}}$$

DNA extraction was carried out using lyophilized *Cyclamen* leaves with the CTAB-based method by Lodhi et al. (1994) with slight modifications. NanoDrop-1000 spectrophotometer (Thermo Fisher Scientific, Waltham, USA) was used for evaluating the purity and concentration of genomic DNA (absorbance ratios 260/280, 260/230).

#### 2.4.1 SRAP analysis

A total of 36 primer combinations were tested, from which 26 combinations showed higher polymorphism levels and were selected for further analysis. Forward and reverse SRAP primer sequences are shown in **Table 1**. For PCR reactions were used 5x green PCR buffer, 0.6 M of each primer (Bioline GmbH, Luckenwalde, Germany), MgCl_2_ (1.5 mM), dNTPs, Taq DNA polymerase (1 U) (Promega, Madison, Wisconsin, USA) and genomic DNA (50 ng). Amplification of DNA was performed in a 96-well Eppendorf Mastercycler Nexus PCR Cycler (Sigma-Aldrich GmbH, Darmstadt, Germany) as described by (Li and Quiros, 2001) and (Simsek et al., 2017) with minor changes: denaturation at 94°C for 5 min, followed by five cycles at 94°C, annealing at 35°C and elongation at 72°C for 60 s, followed by 35 cycles at 94°C, 50°C and 72°C for 60 s with a final elongation at 72°C. Separation of amplicons was performed on agarose gels (2%) (Promega, Madison, Wisconsin, USA) in 1 x TAE, at 0.29 V/cm^2^ for 1.5 hours, and detected by EtBr staining (Sigma-Aldrich GmbH, Darmstadt, Germany), using a 50 bp DNA ladder (GeneDireX, Inc., Taoyuan, Taiwan). PCR amplifications were performed in duplicate.

**Table 1:** Forward and reverse SRAP primer sequences used in the study.

SRAP forward primer	Sequence (5′-3′)	Tm (°C)	GC content (%)
me1	TGAGTCCAAACCGGATA	48.8	47.1
me2	TGAGTCCAAACCGGAGC	54.7	58.8
me3	TGAGTCCAAACCGGAAT	49.8	47.1
me4	TGAGTCCAAACCGGACC	54.4	58.8
me5	TGAGTCCAAACCGGAAG	51.2	52.9
me6	TGAGTCCAAACCGGACA	52.9	52.9
SRAP reverse primer			
em1	GACTGCGTACGAATTAAT	45.6	38.9
em2	GACTGCGTACGAATTTGC	51.3	50.0
em3	GACTGCGTACGAATTGAC	50.4	50.0
em4	GACTGCGTACGAATTTGA	49.0	44.4
em6	GACTGCGTACGAATTGCA	52.5	50.0
ba1	GTCGAGCTGCCAATTATA	48.3	44.4

#### 2.4.2 SCoT analysis

A total of 20 SCoT primers were evaluated, from which 12 generated clear bands and enabled the selection of consistent and rich polymorphisms. SCoT primer sequences are shown in **Table 2**. PCR reaction mixtures comprised Green Master Mix (Promega), 20 mM Tris-HCl, 1.5 mM MgCl_2_, 50 mM KCl, 0.24 mM dNTPs, Taq polymerase (0.5 U) (Promega), primer (0.8 μm) and template DNA (25 ng). Amplification was performed using the protocol described by (Collard and Mackill, 2008), with few modifications: denaturation at 94°C for 3 min, followed by 35 cycles of 94°C, 50°C, and 72°C for 1 min, and a final extension at 72°C for 5 min. PCR amplification products were separated as described in the previous sub-section, using a 100 bp DNA ladder (GeneDireX, Inc. USA).

**Table 2:** SCoT primer sequences used in the study.

SCoT primer	Sequence (5′-3′)	Tm (°C)	GC content (%)
SCoT 1	CAACAATGGCTACCACCA	52.0	50
SCoT 2	CAACAATGGCTACCACCC	53.5	55.6
SCoT 3	CAACAATGGCTACCACCG	53.7	55.6
SCoT 4	CAACAATGGCTACCACCT	51.8	50.0
SCoT 5	CAACAATGGCTACCACGA	52.0	50.0
SCoT 6	CAACAATGGCTACCACGC	54.3	55.6
SCoT 7	CAACAATGGCTACCACGG	53.7	55.6
SCoT 8	CAACAATGGCTACCACGT	52.3	50.0
SCoT 13	ACGACATGGCGACCATCG	58.3	61.1
SCoT 16	ACCATGGCTACCACCGAC	57.5	61.1
SCoT 30	CCATGGCTACCACCGGCG	62.8	72.2
SCoT 33	CCATGGCTACCACCGCAG	59.5	66.7

### 2.5 Data analysis

#### 2.5.1 Molecular marker analysis

Clearly and repeatable SCoT and SRAP segments were evaluated as present (1) or absent (0). The polymorphic bands number (PB) and the ability to reveal dissimilarity in SRAP and SCoT markers were evaluated by calculating the polymorphism, diversity index (H), polymorphic information content (PIC), effective multiple ratio (E), marker index (MI), discriminating power (DP), band informativeness (Ib) and resolving power (RP).

H was calculated as follows:


$$H=1-\sum^{}p_{i}{\;}^{2}$$

where *p_i_* is the allele frequency for the *i* ^th^ allele (Liu, 1998).

PIC of each SRAP and SCoT primers pair was estimated accordingly:


$$\begin{array}{l} PIC=1-\;\left(\sum_{i=1}^{n}p_{i}^{2}\right)-\\ \left(\sum_{i=1}^{n=1}\sum_{j=i+1}^{n}2q_{1}^{2}q_{j}^{2}\right) \end{array}$$

where *n* is allele number (marker), *q_i_* is the *i^th^* allele frequency, and *q_j_* is the *j^th^* allele frequency (Botstein et al., 1980).

The effective multiple ratio (E) was calculated according to (Powell et al., 1996), as follows:


$$E=n\frac{n_{p}}{n_{p}+n_{np\;}}$$

where *p* and *np* represent the polymorphic and non-polymorphic markers fraction.

MI was calculated (Powell et al., 1996; Amiryousefi et al., 2018) as follows:


MI=PIC x PB

DP was calculated as follows:


$$DP=1-\sum^{}p_{i}\;\frac{Np_{i}-1}{n-1}$$

where the *i^th^* pattern of the particular *j^th^* primer, present at frequency *p*_i_ in a set of varieties, and *N* as individuals, according to (Tessier et al., 1999).

The band informativeness (Ib) was calculated accordingly:


$$Ib=1-\left(2\;x\;\left|0.5-p_{i}\right|\right)$$

where *p_i_* is the band frequency amplification (Prevost and Wilkinson, 1999).

RP was calculated as follows:


$$R=\;\sum^{}Ib$$

Additionally, the Shannon Wiener (H′) (Shannon, 1948), Simpson’s Indices (λ) and Pilou evenness (J) were calculated to evaluate the genetic diversity and assessed using library vegan from R (version 4.2.2) (Oksanen et al., 2019).

#### 2.5.2 Multivariate analysis of phenotypic, colorimetric, and genotypic data using hierarchical clustering.

Principal component analysis (PCA) was performed using the FactoMiner factoextra package (Lê et al., 2008). The unweighted pair group method with arithmetic mean (UPGMA) was used to construct hierarchical cluster analysis (HCA) based on similarity matrices (Euclidean distance) between the morphological, colorimetric and genetic analysis. Euclidean distance was estimated using the Cluster R package (Maechler et al., 2019). The dendextend program was used to display tanglegrams, visual approaches for comparing two trees with the identical set of labels connected by lines (Galili, 2015). The entanglement coefficient ranges between 0 (complete entanglement) and 1 (no entanglement), and was used to evaluate the effectiveness of the alignment of the two trees; a lower coefficient corresponds to a good alignment. Using color gradients for dissimilarity determination across individuals, visual representations of matrices for the morphological and molecular data were generated using the corrplot package in R.

## 3 Results

### 3.1 Principal component analysis and clustering pattern based on morphological diversity

**Figure 2:**
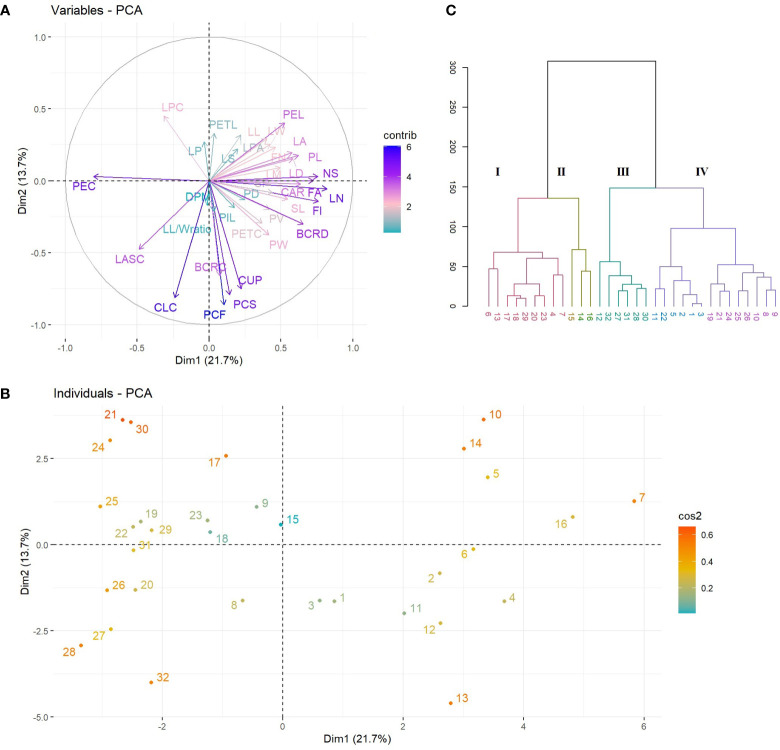
PCA plots of variables **(A)** and species **(B)**. The first two dimensions accounted for 35% of full variance. HCA of *Cyclamen* genotypes based on morphological data (Euclidean distance, r=0.73); each color represents a different cluster).

The PCA results revealed that the first ten PC, with eigenvalues ranging from 1.19 to 7.89, were significant in explaining the variation between the evaluated *Cyclamen* species, accounting for 82.98% of the total variation. (**Supplementary Table S3**; **Figure 2**). The 1^st^ PC accounted for 21.79% variation and pointed out dissimilarities in NS, FI, FN, PL, SL, PEL, PEC, FA, BCRD, and LN, representing traits associated to flower development, along with CAR and CA associated to plant development. The 2^nd^ PC accounted for 13.78% of the variance and correlated with PCF, PCS, BCRC, CUP, CLC and LPC, suggesting the relation with flora color traits. The 3^rd^ component highly correlated with leaf characteristics, mainly LL, LPA and LA and explained 9.76% of the total variance. The 4^th^ and 5^th^ PCs accounted for 9.31% and 6.59% of the total variance, and explained variation in LL, LW, LM, DPM and LP, respectively. Phenotypic variations of the 36 evaluated traits were assessed (minimum, maximum, median, mean, and Kurtosis variation) and are presented in **Supplementary Table S4**. As seen in **Figure 1A**, the first quadrant highlights the LPC in genotypes 21 (*C. mirabile*), 24 (*C. balearicum*), and 25 (*C. cilicium*) with a silver green pattern. The second quadrant highlights samples 7 and 10 that presented the highest pedicel length. The third emphasized the light purple upper corolla of *C. persicum* cv. Petticoat Dark Violet (13). The last quadrant, emphasized the genotypes 27 (*C. coum*), 28 (*C. elegans*) and 32 (*C. purpurascens*) with a red-purple abaxial surface color.

HCA was performed to better discriminate the genotypes based on their morphology. Thus, the grouping pattern of the *Cyclamen* species for morphological diversity using Euclidean distance with UPGMA algorithm for clustering revealed four major clusters (**Figure 2C**). Cluster I (red) highlights four *C. persicum* genotypes (4, 6, 7 and 13) with *C. africanum*, *C. hederifolium*, *C. pseudibericum*, *C. alpinum* and *C. hildebrandii*. These genotypes presented medium canopy architecture, a similar petal color (light carmine), leaf width/width ratio, medium pedicel length and a high plant vigor. Cluster II (brown) highlights genotypes 14, 15 and 16 (Merengue group and Smartiz type) with the highest canopy area, petiole diameter and similar flower area dimensions. Cluster III (green) comprised a single *C. persicum* genotype (12-Verano) and five species, namely *C. coum*, *C. elegans*, *C. intaminatum*, *C. libanoticum*, and *C. purpurascens*. These genotypes presented a light purple color at flowering and a deep purple at senescence, similar color of upper and lower corolla, lower canopy area and the absence of leaf pattern. The largest number of genotypes was identified in cluster IV (purple) with 14 genotypes. Out of them, eight were *C. persicum* accessions comprising the Metis group (1-3, 5), Victoria (9), and Petticoat group (10, 11), whereas the remaining genotypes were *C. balearicum*, *C. cilicium*, *C. confusum*, *C. cyprium*, *C. mirabile*, and *C. mirabile* ‘Tilebarn Nicholas’. Genotypes in cluster IV presented a paler-pink petal color and upper corolla, along with a lower flower number, but a medium to strong degree of lobbing in leaf lamina.

### 3.2 Color distribution among different parts of Cyclamen leaves and flowers

A phenotypic trait that commonly reflects the plants physiological condition is the color of the flowers and leaves. Furthermore, the variation in pigment provides information on the genetic intra- and interspecific taxa and/or populations variability, recognizing the advancement of evolutionary ideas from a phylogenetic perspective. The color indicators of the green, silvery lamina and petiole are presented in **Supplementary Figure 1**, whereas the indicators of the slip, eye, and petiole are presented in **Supplementary Figure 2**. Variation in color parameters were assessed and are presented in **Supplementary Tables S5**, **S6**. In flowers, the luminance L* ranged between 10.0 (eye, C20) and 99.7 (slip, C24). The redness a* between -8.6 in sepal in C6 and 72.8 in C9 (slip) denoting a tendency of red and purple pigment accumulation, and the yellowness b* with the lowest value of -39.3 (C28) in slip and the highest in sepal 43.8 in C30. Regarding the leaves, the L* ranged between 12.9 (C5) and 87.6 (C24). The redness a* varied between -32.2 (C7) and 33.8 (dark pattern, C22), whereas b* ranged between -8.2 (C22) and 42.6 (petiole of C18).

**Figure 3:**
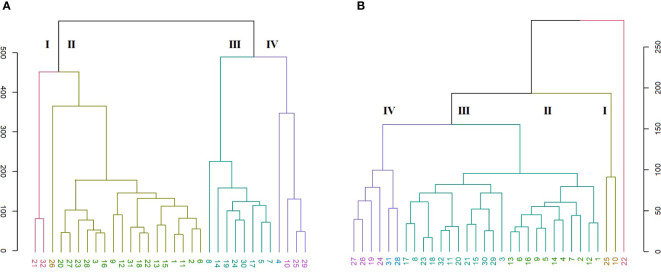
HCA of color characteristics of *Cyclamen* genotypes flowers (A; *r*=0.92) and leaves (B; *r*=0.96).

The grouping pattern of *Cyclamen* flowers (**Figure 3A**) revealed four major clusters, where the first cluster (red) grouped *C. mirabile* (21) and *C. purpurascens* (32). These genotypes presented similarities in terms of L* and hue in the upper part of the petal (slip), a* and hue in sepal and b* and hue in petiole. The following cluster (brown) comprised the largest number of genotypes which presented similar a* and b* values in slip, higher hue level in the lower part of the petal (eye), higher a*, lower b* in sepal and lower hue in petiole. The subsequent cluster (green) comprised lighter-colored samples as seen by the higher values in L*. The last cluster (purple) highlighted two *C. persicum* genotypes, *C. cilicium* and *C. hildebrandii*. These genotypes had the lowest values in hue angle and the lowest in a* and b* in the eye of flower, and similar b* values in flower pedicel. The last two clusters outlined the dark red *C. persicum* genotypes (8 and 4), that were closely followed by the grey-white genotypes 10 and 14 due to their distinct color characteristics compared with the other genotypes with pink and purple color patterns.

The *Cyclamen* leaves (**Figure 3B**) were organized into two major clusters except *C. mirabile* ‘Tilebarn Nicholas’ (22), considered an ‘outlier’ due to its distinct purple-green color pattern. The first major branch (cluster I, brown) includes genotypes *C. persicum* ‘Petticoat pure white’ (10) and *C. cilicium* (25) with similar L*, b* and C* of the green and light-green patterns. Subsequently, cluster II (olive) comprised the majority of *C. persicum* genotypes, with similarities in a* and hue in darker green lamina, together with b*, C* and hue values in silvery lamina. The following cluster III (green), mainly grouped the genotypes with similar a* (darker green lamina), chroma and shade in leaves, along with similarities in terms of petiole hue. The last, cluster IV (purple) emphasized *C. elegans* (28) and *C. libanoticum* (31) that presented similar color in petiole and in light silver and green patterns. *C. cyprium* (19), *C. balearicum* (24), *C. confusum* (26), and *C. coum* (27) presented similar petiole color characteristics in terms of L*, a* and C*.

### 3.3 Summary statistics and clustering pattern of Cyclamen genotypes based on molecular diversity indices

#### 3.3.1 SRAP analysis

**Figure 4:**
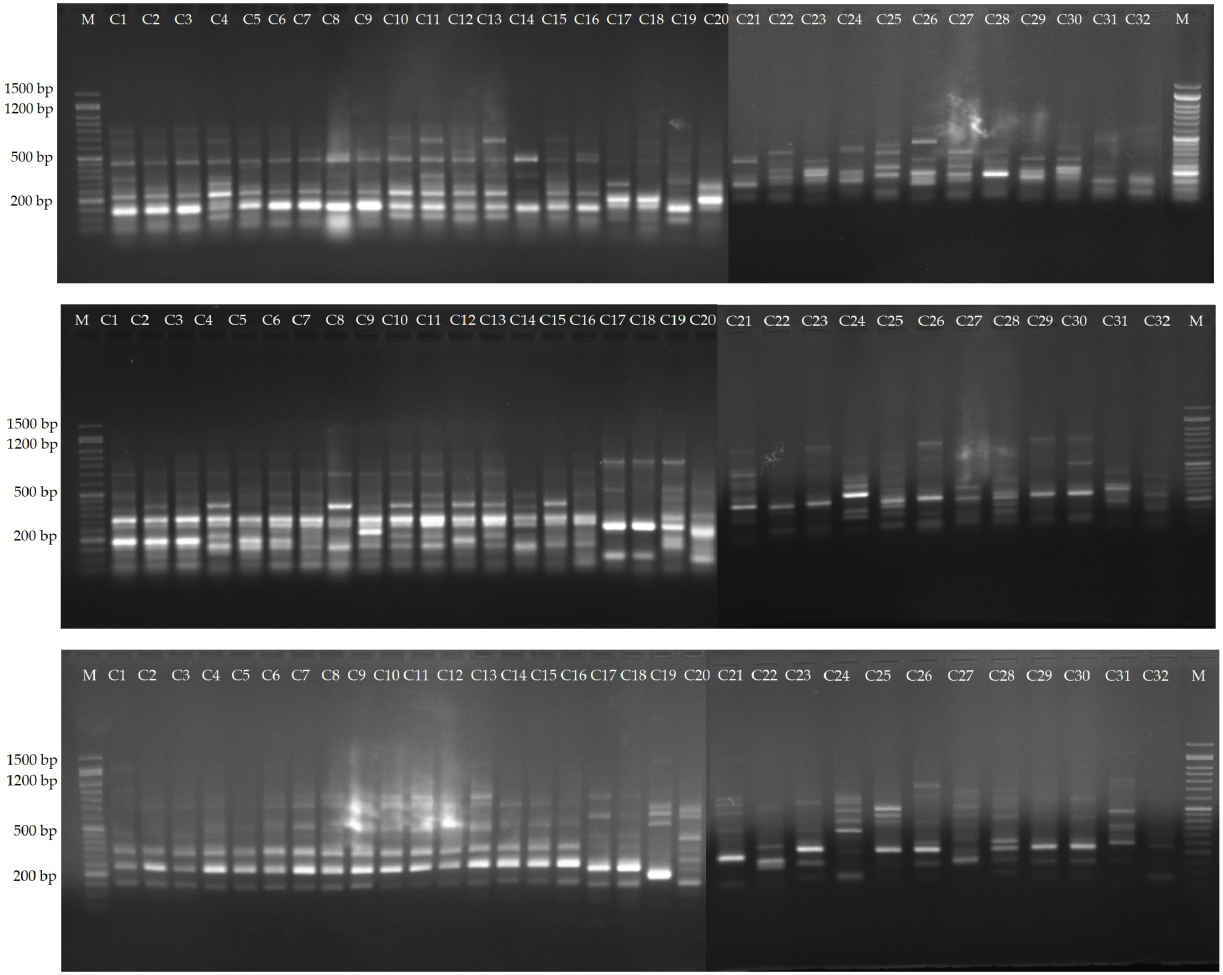
Amplification profiles of markers me1-em4, me1-em6 and me2-em3. Lane 1-16, *C. persicum* cultivars, 16-32, different *Cyclamen* species.

Among the evaluated 36 primer combinations, 26 produced higher polymorphism, with a total of 349 bands of which 264 showed higher polymorphism. The polymorphic bands per primer combination ranged from 5 (me1-em3) to 15 (me2-em4, me4-em6 and me4-em6), with an average of 10.1. The percentage of PB in each primer varied between 56.25 and 88.23%, with an average of 75.0%. Three representative profiles (me1-em4, me1-em6, me2-em3) are shown in **Figure 4**.

Indicators including H, PIC, E, MI, D, and R were used to evaluate the polymorphisms and discriminatory capacity of the tested markers. H had an average value of 0.30 with the highest observed in me1-em2. The mean values of E, MI, D and R were 2.91, 2.78, 0.93 and 5.35, respectively (**Supplementary Table S7**).

#### 3.3.2 SCoT analysis

**Figure 5:**
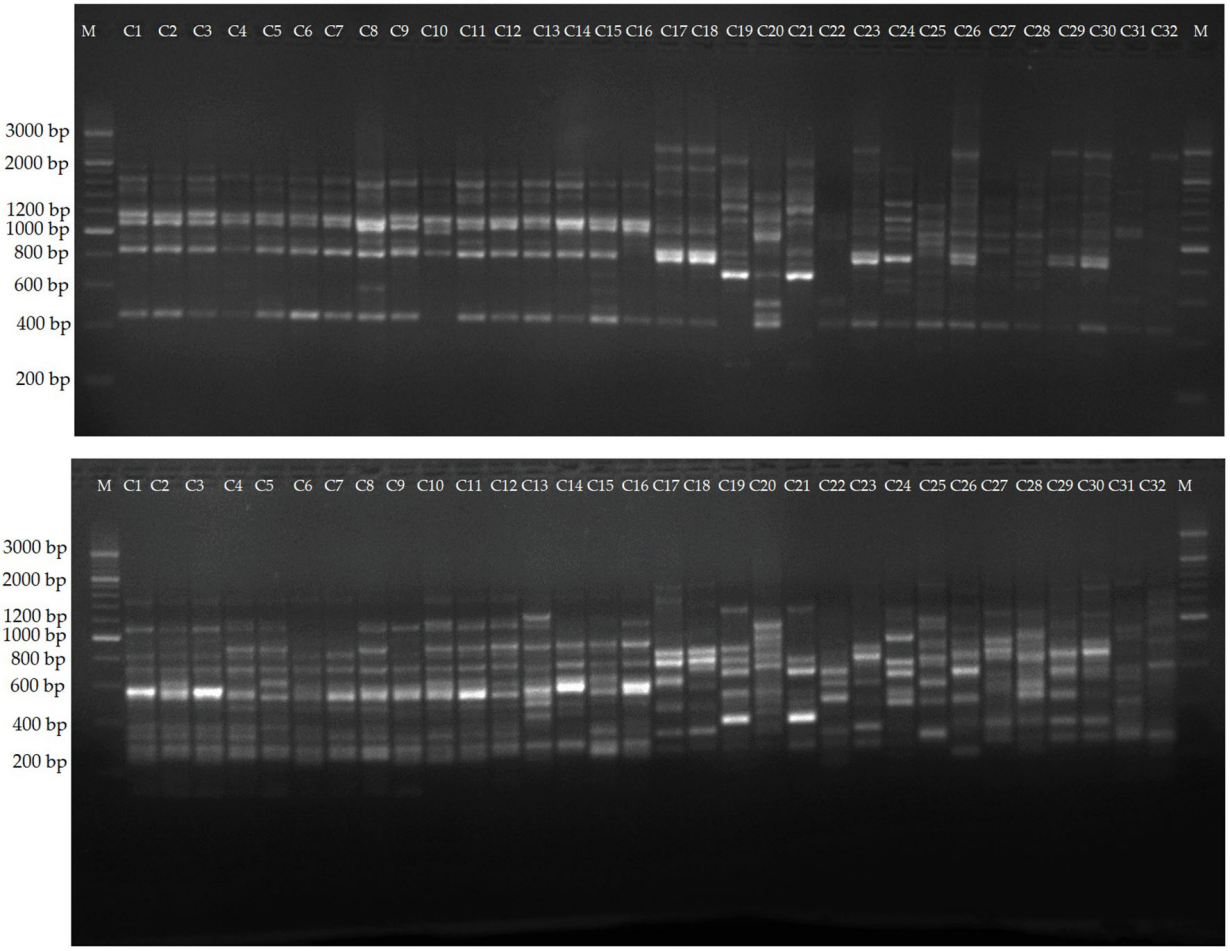
Amplification profiles of markers SCoT 6, and SCoT 13. Lane 1-16, different *C. persicum* genotypes, 16-32, different *Cyclamen* species.

The selected 12 primers generated 204 reliable SCoT bands, from which 169 proved to be polymorphic. The PB per primer ranged from 4 (SCoT5) to 19 (SCoT13 and SCoT30), with an average of 14.1. PB percentage varied between 71.42 and 90.0%, with a mean value of 82.43%. Two representative profiles (SCoT 6, and SCoT 13) are shown in **Figure 5**. The mean value of H was 0.38, with a range between 0.499 in SCoT4 and 0.173 in SCoT5. PIC had a mean value of 0.33 with the highest in SCoT 2. The average values of E, MI, D and R were 3.19, 4.93, 0.93 and 4.35, respectively (**Supplementary Table S8**).

**Figure 6:**
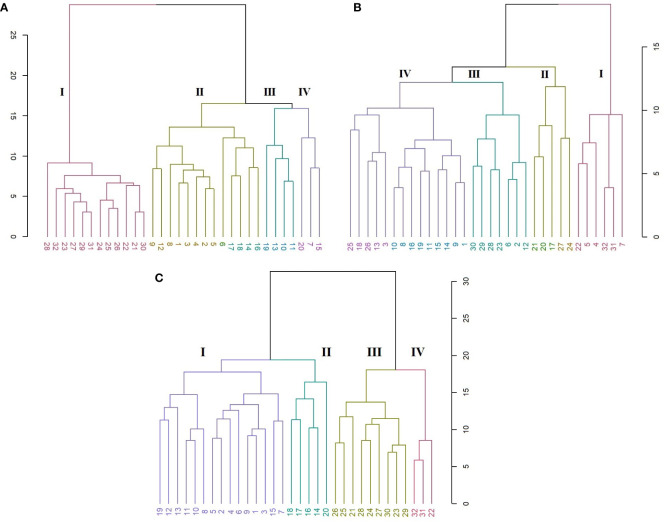
HCA representing the genetic relationships among the 32 *Cyclamen* genotypes from the SRAP (**A**; *r*=0.95), SCoT (**B**; *r*=0.82) and combined (**C**; *r=*0.93) analysis. Each color represents a different cluster.

In the SRAP analysis, the HCA organized the genotypes in four main clusters (**Figure 6A**). A clear discrimination is seen between the *C. persicum* genotypes and other *Cyclamen* species. Cluster I (red) comprised *C. elegans* (28) as an ‘outlier’, mainly due to its silvery patterned leaves, closely followed by *C. purpurascens* (32), *C. alpinum* (23), *C. coum* (27), *C. x hildebrandii* (29) and *C. libanoticum* (31) with pink colored flowers, magenta blotches and silver speckled leaves. This cluster also comprised *C. mirabile* (21)*, C. mirabile* ‘Tilebarn Nicholas’ (22) and *C. intaminatum* (30), with similar flowers and lower corolla diameter, followed by *C. balearicum* (24)*, C. cilicium* (25), and *C. confusum* (26), with similar white to pinkish flowers and morphology. Cluster II (brown) consisted of Metis and red-colored *C. persicum* types. Cluster III (green) comprised the Petticoat types (10, 11, 13) and *C. cyprium* (19). The last cluster (IV, purple) included two *C. persicum* genotypes (7 and 15) and *C. pseudibericum* (20).

The SCoT analysis organized the genotypes mostly by their color characteristics, but also on their habitat as described by breeding companies (**Figure 6B**). Thus, cluster I (red) comprised genotypes 22 (*C. mirabile* ‘Tilebarn Nicholas’), 31 (*C. libanoticum*), and 32 (*C. purpurascens*), probably due to their distinct leaf color pattern (22 and 32), redish eye and pink-colored petal margins. The *C. persicum* genotypes 4, 5, 7 presented dissimilar flower characteristics and leaf color, but proved to be resistant to *Botrytis cinerea* (gray mold). Cluster II (brown) comprised *C. pseudibericum* (20) and *C. coum* (27), with large carmine flowers,white rim and a dark stain around the mouth and leaves marked with a hastate pattern, speckling, or blotching of silver and green. Genotypes *C. africanum* (17), *C. mirabile* (21), and *C. balearicum* (24) presented similarities in flowers with white to pale pink. Furthermore, these genotypes bloom prior to leaf emergence particularly in autumn and spring. Cluster III (green) comprised genotypes *C. alpinum* (23), *C. elegans* (28) with pink flowers, a deep magenta blotch and round leaves with silver markings, along with *C. x hildebrandii* (29), *C. intaminatum* (30) and *C. persicum* accessions 2, 6, and 12 with pink hues. Genotypes 2, 6 and 12 are resistant to both winter and summer conditions and according to breeding companies exhibit outstanding outdoor performances. Cluster IV (purple) comprised the Merengue and Petticoat *C. persicum* accessions, with fringed and fragrant flowers. Furthermore, genotypes 1, 9, 14 and 15 are resistant to high temperatures. The following *C. hederifolium* (18), *C. cilicium* (25) and *C. confusum* (26) with similar flower colors and leaf patterns, bloom in the winter and are resistant to rainy days.

In order to obtain more accurate genetic estimates, combined analysis was carried out using all molecular data (**Figure 6C**). The dendrogram grouped the individuals into four main clusters. Cluster I (red) comprised genotypes 22, 31 and 32 similar to SRAP analysis. Clutser II (olive) organized the genotypes that are part of the subgenus *Gyrophoebe*, except *C. balearicum* (24) (subgenus *Psilanthum*). Cluster III (green) comprised genotypes *C. africanum* (17) and *C. hederifolium* (18) part of subgenus *Cyclamen* together with two *C. persicum* accessions (Merengue type) and *C. pseudibericum* (20) that corresponds to subgenus *Gyrophoebe*. Cluster IV (purple) comprised *C. persicum* genotypes with Metis and Smartiz types in the same sub-cluster followed by the Petticoat types in the following sub-cluster.

**Table 3** displays the most frequently used indexes calculated for 32 *Cyclamen* genotypes based on morphological and molecular data. The Shannon-Wiener index was generally high in the SRAP analysis, with the highest value in *C. libanoticum* (31) and the lowest in *C. persicum* accession Smartiz (15), along with the color characteristics that had the highest value in *C. persicum* Metis Vitoria (2) and the lowest in *C. purpurascens* (32). The combined molecular data revealed the highest *H’* value in *C. persicum* Jive (8) and the lowest in *C. purpurascens*. Comparatively, the phenotypic data and SCoT analysis presented relatively close lower values. The same similarities were observed in the Simpson’s indices. Conversely, the Pilou evenness index was generally high in color parameters in *C. purpurascens* (32) with the lowest values observed in *C. hederifolium* (18).

**Table 3:** Genetic diversity indices based on phenotypic, color and genotypic data of *Cyclamen* genotypes.

	Phenotypic data				Color characteristics				Genotypic data											
									SRAP markers				SCoT markers				Combined data			
Diversity indices	Min	Median	Max	Average	Min	Median	Max	Average	Min	Median	Max	Average	Min	Median	Max	Average	Min	Median	Max	Average
**Shannon–Wiener Index (H′)**	1.467	2.247	2.96	2.24	1.9869	3.2604	3.3142	2.9022	1.9869	3.2604	3.314	2.902	2.04134	2.32273	2.4575	2.3	2.707	3.577517	3.646	3.3483
**Simpson’s Index (λ)**	0.4876	0.7575	0.919	0.742	0.8512	0.9583	0.9583	0.9328	0.8512	0.9584	0.9617	0.932	0.8531	0.896	0.9125	0.89	0.9259	0.969894	0.9726	0.9593
**Pilou evenness (J)**	0.1382	0.21368	0.2677	0.211	0.28843	0.2855	0.4093	0.3184	0.1323	0.2046	0.2695	0.2045	0.2613	0.2645	0.3268	0.28	0.2613	0.264574	0.3268	0.2796

**Figure 7:**
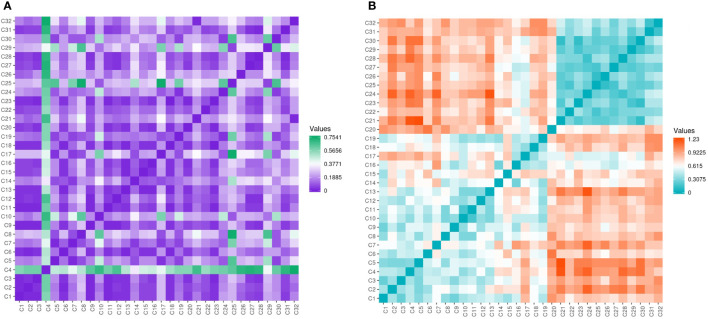
Gower’s dissimilarity matrix from the morphological data **(A)** and Pearson’s correlation matrix **(B)** from the molecular data of the *Cyclamen* genotypes. For the morphological data, green represents similarities, while purple represents dissimilarities. For the molecular data, orange represents similar genotypes, whereas turquoise represents the most dissimilar genotypes. As the matrices were symmetrical, the values below the crossways correspond to the values above.

Assessment of genetic data revealed low variability among the evaluated genotypes with few exceptions’ seen by the different color gradient (**Figure 7A**). Conversely, the genetic data was high among *Cyclamen* genotypes and lower among *C. persicum* accessions as revealed by the higher ginger squares number in **Figure 7B**.

#### 3.3.3 Genetic diversity using linkage analysis for morphological, colorimetric and molecular data

**Figure 8:**
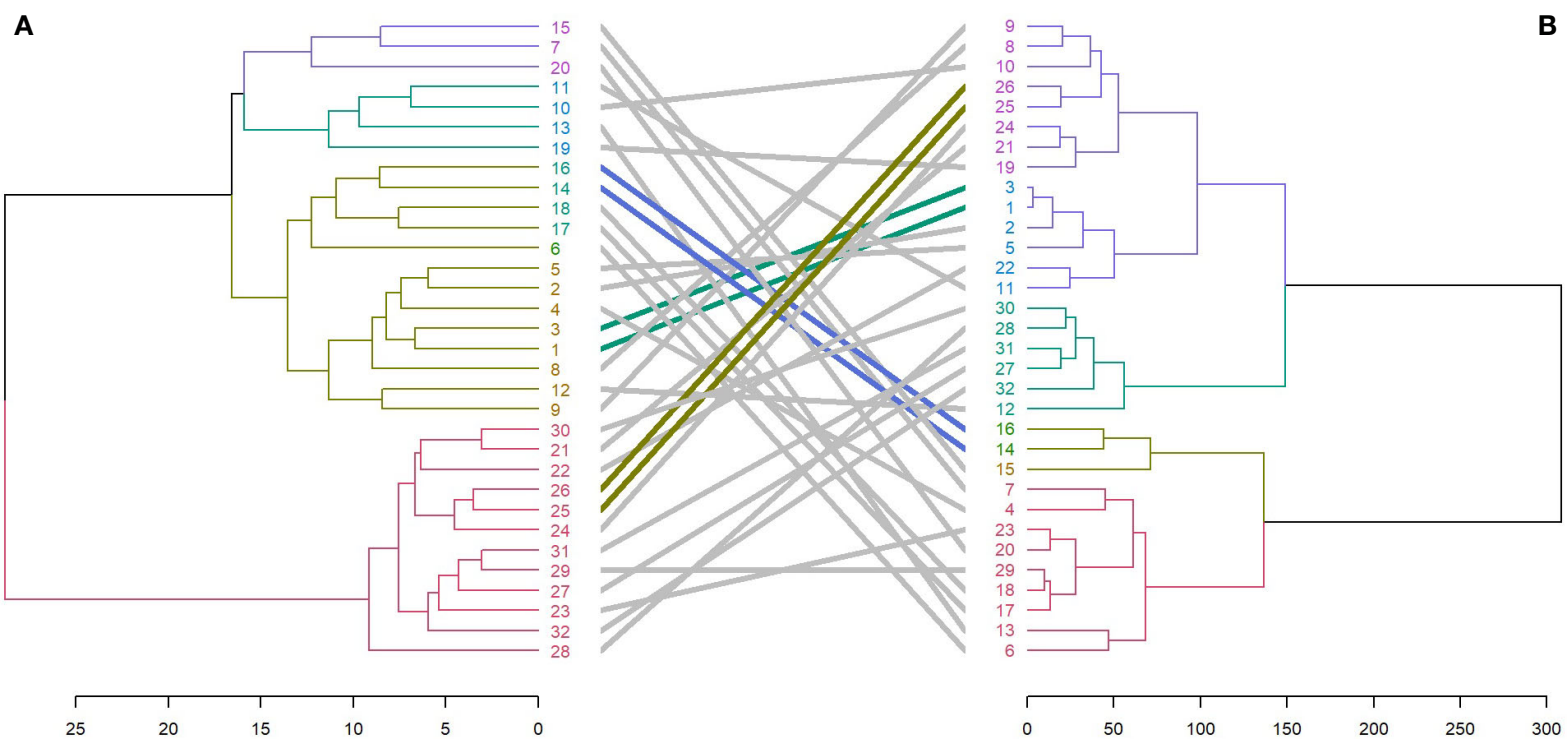
Comparison of dendrograms for the 32 *Cyclamen* genotypes from SRAP markers **(A)**, and phenotypic data **(B)** with entanglement = 0.30. The grey lines in connecting the dendrograms correspond to mismatched genotypes whereas the colored lines are genotypes that maintained their position between clusters.

Entanglement analysis (0.30) among dendrograms obtained from the morphological and SRAP anlayses showed a high association. The highest similarities in joint analysis of morphological data and SRAP analysis were observed in accessions 1 and 3, Merengue types 14 and 16, along with *C. cilicium* (25) and *C. confusum* (26). Accession 6 (Halios falbala) along with *C. africanum* (17) and *C. hederifolium* (18) maintained their position in the same cluster with slight order modifications (**Figure 8**).

The HCA generated from the phenotypic data were compared with the color characteristics. Higher similarities were noticed when separating the colorimetric data by flowers and leaves. In terms of color characteristics of leaves and phenotypic data (entanglement=0.49), two genotypes maintained the same position in the cluster as seen by the colored lines between the dendrograms in *C. persicum* Halios falbala (6) and Petticoat dark violet (13). Furthermore, similarities were also noticed in Merengue types (4, 7, 15 and 16) that grouped in the same sub-cluster even though not in the same order (**Supplementary Figures 3A, B**). Most similarities to SCoT analysis were noticed in the colorimetric data for leaves, (entanglement=0.57) in *C. x hildebrandii* (29) and *C. intaminatum* (30) (**Supplementary Figures 3C, D**), and flowers, respectively, where two *C. persicum* accessions (2 and 6) maintained the same cluster position (**Supplementary Figures 4A, B**). Also, genotypes *C. alpinum* (23), and *C. elegans* (28) presented similarities and were grouped in the same cluster even though not in the same order. The HCA generated from the colorimetric analysis of *Cyclamen* flowers compared with SRAP data (entanglement=0.55) presented similarities in two *C. persicum* accessions (9 and 12) which maintained in the same cluster positioning. Genotypes *C. alpinum* (23)*, C. coum* (27)*, C. elegans* (28), and *C. purpurascens* (32) grouped in the same cluster with small differences in their position (**Supplementary Figures 4C, D**). The grouping pattern of the molecular markers used (SRAP and SCoT) presented low similarities with no entanglement (data not shown), which is not unexpected as these primers are not specific for *Cyclamen*.

## 4 Discussion

Prior research has found considerable morphological differences between closely related accessions or populations, due to the climatic conditions of various habitats, and lower heredity of reproductive and vegetative traits in *Cyclamen* (Yesson and Culham, 2011; Cornea-Cipcigan et al., 2022b). In the present study, PCA was performed to assess the similarities and differences between genotypes using morpho-agronomic characters. PCA generally supported the assemblage of phenotypic traits into species-specific clusters, as seen by *C. persicum* genotypes that mainly grouped in the 2^nd^ and 3^rd^ quadrants. Significant variables composing PC1 were NS, PL, FA, LN, CA, and BCRD as seen by the highest eigenvalues, whereas PC2 accounted for the color characteristics of flowers (**Supplementary Table S3**). Using the 36 morphological parameters from the first ten PCs, the UPGMA grouped the genotypes based on their canopy architecture and plant vigor in cluster I, lower canopy area and absence of leaf pattern in cluster II. Highest canopy area and similar flower area dimensions were grouped in cluster III, whereas the IV^th^ cluster comprised paler pink colored genotypes with lower flower number, and strong degree of lobbing. These are in accordance with morphological descriptions of different *Cyclamen* species, as the most distinctive variables were found to be quantitative with the highest eigenvalues in the first PCs (Curuk et al., 2015). Several researchers claim that morphological characters are less effective for assessing genetic diversity owing to the shift in environmental conditions and plant growth development; HCA groups the samples mainly based on their morphological characters rather than geographical origin (Shinwari et al., 2014; Curuk et al., 2015; Curuk et al., 2016). Nonetheless, morphological and agronomic characterization is significant for describing plant populations and for selecting varieties with favorable traits for crop development that is of great interest for plant breeders. Other classifications, such as chromosome number, duration of green period, early or late blossoming period and flowering habit, disease spot detection and fruit color were successfully assessed in different ornamental and medicinal crops that may demonstrate more complex distribution classification (Chaudhary et al., 2012; Cui et al., 2019; Lu et al., 2021).

A phenotypic trait that often reflects the physiological status of the plant is the color of the flowers and leaves (Noman et al., 2017; Cui et al., 2019). The concentration of secondary metabolites with considerable chromatic elements, that may have decorative, medicinal, and economic relevance, is indicated by the color of the flowers and leaves (Cornea-Cipcigan et al., 2022a). Color variation also gives data on the genetic inter- and intraspecific diversity of populations and/or species, recognizing the progress of evolutionary principles from a phylogenetic standpoint (Tanaka et al., 2008; Klančnik et al., 2016). Color parameters were assessed to evaluate the color of *Cyclamen* flowers and leaves that may aid in differentiating similar varieties or species. Genotypes that presented higher values in L* grouped in the same cluster (cluster II, **Figure 3A**). Furthermore, distinct color characteristics were observed in dark red and grey-white genotypes that grouped in the same cluster (III and IV, **Figure 3A**). Regarding the leaves, *C. mirabile* ‘Tilebarn Nicholas’ (C22) is considered an ‘outlier’ due to its purple-green leaf pattern. Genotypes *C. persicum* ‘Petticoat pure white’ (10) and *C. cilicium* (25) presented similar L*, b* and C* in the dark and silver patterns (cluster I, **Figure 3B**). *C. persicum* genotypes presented similarities in a* and hue in dark lamina and b* and C* in silver pattern, whereas the following cluster comprised genotypes with similar a* (darker green lamina), C* and shade in leaves, together with similarities in terms of petiole hue (II and III, **Figure 3B**). The genotypes with similar petiole color characteristics grouped in cluster IV. Similar findings were observed in *C. purpurascens*, where semi-silvery and patterned leaves had higher L* values compared with green and silver lamina. According to the study’s findings, all genotypes showed positive b* values, indicating that the leaves had a faint yellow tint (Osterc et al., 2014; Cornea-Cipcigan et al., 2019). Additionally, higher hue levels were noted in *C. purpurascens* flowers and lower values in the leaves (Osterc et al., 2018). Consistent with previous reports, the values of b* were significantly lower in dark-colored flowers and higher in leaves (Cui et al., 2019).

Genetic diversity analysis using the Euclidean distance generated with the SRAP (*r =* 0.95) and SCoT (*r=* 0.82) markers partitioned the *Cyclamen* genotypes into four groups. This study firstly evaluated the effectiveness of SCoT markers to determine genetic variation between *Cyclamen* genotypes. SCoT markers have been chosen based on their successful use for analysis of genetic diversity and cultivar identification in multiple plants. A relatively high percentage of polymorphic bands (82%) was detected, consistent with the proportion of polymorphism reported in other medicinal and ornamental species, such as sage (Etminan et al., 2018), chrysanthemum (Feng et al., 2016) and orchids (Tikendra et al., 2021). In the SRAP analysis, a high percentage of polymorphic bands (75%) and a clear discrimination between genotypes was observed, as shown by the high cophenetic correlation coefficient (*r*=0.95). SRAP primers have been chosen due to their high reproducibility compared to RAPDs and ease of assay performance than AFLPs. Previously, UPGMA dendrograms were constructed to assess the similarities and differences between *Cyclamen* species form different regions of Turkey showing a clear discrimination between *C. persicum* genotypes and other species. Furthermore, *C. pseudibericum* and *C. alpinum* genotypes clustered in different groups based on their geographic origin, demonstrating their narrow genetic diversity (Taşkin et al., 2012; Simsek et al., 2017). Consistent with previous studies on SRAP markers (Simsek et al., 2017), *C. hederifolium* and *C. persium* clustered in the same group (**Figure 6A**). Furthermore, *C. intaminatum* and *C. cilicium* grouped in the same sub-cluster, while *C. alpinum* was closely followed by *C. coum*, possibly due to the same chromosome number (2n = 30). These genotypes are genetically related as already reported (Compton et al., 2004). The combined SRAP and SCoT analysis revealed a more accurate genetic estimate between the genotypes. For example, the 2^nd^ cluster organized the genotypes that mainly correspond to subgenera Gyrophoebe with few exceptions, whereas 3^rd^ cluster comprised genotypes corresponding to *Cyclamen* subgenus. This is in accordance to previous studies that revealed a similar phylogeny using cluster analysis (Anderberg et al., 2000; Compton et al., 2004).

Plants that present similarities in terms of agro-morphological characters may have significantly different molecular characteristics, and vice versa. Discrepancies in genotypic and phenotypic data revealed in clusters might be due to the effects of environment-genotype interaction often noticed in quantitative inherited characteristics. Understanding the nature and degree of genetic variety in crops requires non-overlapping and complementary morphological and molecular data that are highlighted by the absence of relationship between the diversity matrices (grey lines). Several researches investigated the discrepancies between phenotypic and genotypic characteristics, such as maize (Hartings et al., 2008), wheat (Soriano et al., 2016), sweet sorghum (Da Silva et al., 2017), kalanchoe (Al-Khayri et al., 2022), sesame (Stavridou et al., 2021). An entanglement rate of 0.65 was obtained among 77 passion fruit genotypes showing dissimilarities between the molecular and agronomic data (Preisigke et al., 2020). The same divergence in sample distribution in dendrograms was identified in 65 hot pepper accessions using genetic and agronomic data (Moreira et al., 2018). The dendrogram’s irregular genotype ordering might be explained by the lack of phenotypic trait specificity of the used molecular markers. They amplify more stable parts of the genome but are unable to relate a genetic marker to a particular phenotypic characteristic.

The present study assessed the variation between clustering methods based on phenotypic and molecular markers (SRAP and SCoT markers) which have not been employed in *Cyclamen*. Several HCA for evaluating genetic diversity in *Cyclamen* were compared, followed by combined analysis using morphological and molecular data. With a few outliers for morphological data (*r* = 0.73), high cophenetic correlation coefficients were noticed for the others. UPGMA method was demonstrated to generate the highest coefficient values for the majority of matrices between morphological, colorimetric, and molecular data, implying that these matrices and distances are well represented as dendrograms. In line with the current study’s findings compared to other methods, the UPGMA clustering approach was shown to provide significant correlation coefficients for genetic diversity investigations in safflower (Houmanat et al., 2021), yam (Granato et al., 2018), sweet potato (Paliwal et al., 2022), and gladiolus (Singh et al., 2018).

To evaluate genetic diversity in plant populations, a method that creates a single matrix from the phenotypic and genotypic dissimilarity matrices was presented (Da Silva et al., 2017). Combined analysis of morphological and molecular data particularly with SRAP markers, showed the highest performance and similarities, whereas SCoT markers and color characteristics demonstrated moderate similarities. For a better classification of genotypes, more complex and significant correlations may be revealed using molecular approaches. However, evaluation of diversity using only molecular markers is insufficient. As a result, the molecular assessment of genetic diversity may adequately describe genetic variability, when combined with phenotypic variance. In *Cyclamen*, genetic biology advances have permitted the development of correlations between molecular markers and morphological characteristics (Yesson and Culham, 2006; Naderi et al., 2009). In this aspect, the combination of agronomic parameters, color characteristics and molecular markers is efficient for assessing genetic diversity across and among species with greater precision, taxonomic study, and genotyping.

## 5 Conclusions

Color indicators of leaves, petioles, flowers and pedicel were measured separately for color phenotypic data. The present study also attempted to establish a more precise characterization of leaf and flower color in order to be used as a framework for *Cyclamen* breeding. Furthermore, this study firstly evaluated the potential of SCoT markers for analyzing genetic diversity among *Cyclamen* genotypes. SRAP markers proved to be useful tools for the separation of *Cyclamen* species and/or genotypes, particularly when combined with phenotypic data, as seen by the high entanglement analysis (0.30) among dendrograms. Conversely, similarities were noticed in the colorimetric data for leaves and SCoT data (entanglement=0.47), compared with the colorimetric analysis of flowers and SCoT markers (entanglement=0.45). Thus, combined HCA of phenotypic and molecular data, particularly with SRAP markers, showed the highest performance and similarities, whereas SCoT markers and color characteristics demonstrated moderate similarities. As a result, we underline that SCoT markers along with color characters are suggested for the characterization of germplasm banks with a large number of genotypes in order to detect duplicate accessions since they are more efficient and economically feasible. Conversely, phenotypic data along with SRAP markers more accurately describe in terms of genotype evaluation for later use in breeding programs.

## Data availability statement

The original contributions presented in the study are included in the article/**Supplementary Material**. Further inquiries can be directed to the corresponding authors.

## Author contributionss

MC-C, RM and CS: conceptualization. CS and RM: methodology. MC-C: software. MC-C: formal analysis. MC-C and CS: investigation. CS and RM: resources. MC-C, CS and RM: writing—original draft preparation. MC-C, CS, RM and DP: writing—review and editing. DP: supervision. RM and CS: funding acquisition. All authors contributed to the article and approved the submitted version.
