# Supplementary material for: Characterization of Cyclamen genotypes using morphological descriptors and DNA molecular markers in a multivariate analysis
Source: Front Plant Sci. 2023 Jan 26;14:1100099. doi: 10.3389/fpls.2023.1100099 (PMC9909266; doi:10.3389/fpls.2023.1100099)
Supplement: Supplementary file 1 [file DataSheet_1.docx]

Supplementary Material

**Supplementary Table S1. Details of the Cyclamen genotypes used in the present study**

| Sample ID | Species | Variety name | Breeding Company | Characteristics | Variety code |
| --- | --- | --- | --- | --- | --- |
| C1 | *C. persicum* | Metis Origami | S.A.S. Morel Diffusion | Heat resistant | 4795 |
| C2 |  | Metis Victoria | S.A.S. Morel Diffusion | Low sensitivity to Botrytis, cold resistant and outstanding outdoor conditions | 4701 |
| C3 |  | Metis Silver leaf | S.A.S. Morel Diffusion | Cold resistant and outstanding garden performance | 4795 |
| C4 |  | Merengue Salmon red | Schoneveld Breeding | Moderate heat resistant | L02013 |
| C5 |  | Metis Blank pur | S.A.S. Morel Diffusion | Low sensitivity to Botrytis and cold resistant | 4120 |
| C6 |  | Halios Falbala | S.A.S. Morel Diffusion | Moderate heat resistant, contained growth even in warm days and optimal choice for autumn | 22650 |
| C7 |  | Latinia Pipoca | S.A.S. Morel Diffusion | Resistance to Botrytis and to moderate heat in Californian climate type | 12590 |
| C8 |  | Jive Salmon Red | Schoneveld Breeding | Moderate heat resistance | L01813 |
| C9 |  | Smartiz Victoria | S.A.S. Morel Diffusion | Moderate heat resistant and excellent outdoor performance | 6701 |
| C10 |  | Petticoat pure white | Schoneveld Breeding | Cold-resistant and reversed flower shape | L02524 |
| C11 |  | Petticoat light eye | Schoneveld Breeding | Cold-resistant and reversed flower shape | L02514 |
| C12 |  | Verano st. | Schoneveld Breeding | Cold and moderate heat resistant | L08885 |
| C13 |  | Petticoat dark violet | Schoneveld Breeding | Cold-resistant and reversed flower shape | L02509 |
| C14 |  | Merengue Pure white | Schoneveld Breeding | Heat resistant and fringed flower shape | L02007 |
| C15 |  | Smartiz Violet Fonce | S.A.S. Morel Diffusion | Low heat resistant and great outdoor performance in Mediterranean climate type | 6095 |
| C16 |  | Merengue Magenta | Schoneveld Breeding | Heat resistant and fringed flower shape | L01991 |
| C17 | *C. africanum* | | Botanic Gardens and Arboretum of Mendel University of Agriculture and Forestry from the Czech Republic, Brno | Cold resistant with flowering before leaf apparition | n.a. |
| C18 | *C. hederifolium* | | Czech Republic, Brno | Moderate heat resistant and excellent outdoor performance particularly with late summer rains | n.a. |
| C19 | *C. cyprium* | | Czech Republic, Brno | Cold resistant with late autumn to winter flowering and extremely fragrant | n.a. |
| C20 | *C. pseudibericum* | | Friedrich Schiller Universität Jena, Institut für Ökologie und Evolution, Botanischer Garten Jena | Very resistant to cold with fragrant flowers; Given the Royal Horticultural Society's Award of Garden Merit | n.a. |
| C21 | *C. mirabile* | | Botanischer Garten Jena | Moderate cold resistant with autumn flowering | n.a. |
| C22 | *C. mirabile* 'Tilebarn Nicholas' | | Plant World Seeds | Very resistant to cold (temperatures as low as -16ºC) in dry conditions | n.a. |
| C23 | *C. alpinum* | | Plant World Seeds | Fragrant flowers with blooming from late autumn to early spring | n.a. |
| C24 | *C. balearicum* | | Plant World Seeds | Cold resistant with fragrant spring blooming flowers | n.a. |
| C25 | *C. cilicium* | | Plant World Seeds | Very resistant to cold, if frosted leaves generally recover | n.a. |
| C26 | *C. confusum* | | Plant World Seeds | Cold resistant with flowering in winter periods | n.a. |
| C27 | *C. coum* | | Plant World Seeds | Cold resistant with flowering from January onwards | n.a. |
| C28 | *C. elegans* | | Plant World Seeds | Sensitive to heat and grows best in cold and dry areas | n.a. |
| C29 | *Cyclamen x hildebrandii* | | Plant World Seeds | Moderate heat tolerant and is a registered hybrid in 1955 of *C. hederifolium* and *C. africanum* | n.a. |
| C30 | *C. intaminatum* | | Plant World Seeds | Winter flowering and excellent outdoor performance | n.a. |
| C31 | *C. libanoticum* | | Plant World Seeds | Very resistant to cold, tolerating temperatures as low as -12ºC in a sheltered drained spot, but sensitive to wet conditions | n.a. |
| C32 | *C. purpurascens* | | Plant World Seeds | Extended blooming time, keeping its foliage throughout summer | n.a. |

**Supplementary Table S2.** List of phenotypic variables assessed for the diversity analysis of the 32 Cyclamen genotypes.

| Variables | Abbreviation | Collection time | Method Used |
| --- | --- | --- | --- |
| Number of stems per plant | NS | 5 months after emergence | Number of stems per plant was counted and recorded |
| Petiole diameter | PD | 6 months after emergence | Measured using ImageJ software |
| Petiole color | PETC | at emergence | visual examination (scoring) 1=white; 2=pale yellow-brown; 3=yellow-green; 4=light pink; 5=light carmine; 6=light purple; 7=deep purple; 8=green-brown |
| Plant vigor | PV | 2 months after emergence | Visual scoring as: 1=weak; 2=medium; 3=vigorous |
| Flowering intensity | FI | at flowering | visual examination (scoring) 0=no bud; 1=aborted bud; 3=low; 5=moderate; 7=abundant; 9=extremely abundant |
| Petal length (mm) | PL | In full bloom | <15= small; 15-25=medium; >25=large; Measured using ImageJ software |
| Petal width (mm) | PW | In full bloom | <3= small; 3-10=medium; >10=large; Measured using ImageJ software |
| Flower area | FA | In full bloom | Measured using ImageJ software |
| Petal color | PCF | at flowering | visual examination (scoring) 1=white; 2= white-pink; 3=pale-pink; 4=deep pink; 5=carmine; 6=light purple; 7=deep purple |
| Petal color | PCS | At senescence | visual examination (scoring); 1=grey white; 2 =diffuse-pink; 3=pale-pink; 4=deep pink; 5=carmine; 6=light purple; 7=deep purple; 8=dark pink; 9=dark red |
| Basal corolla ring color | BCRC | at flowering | visual examination (scoring) 1 =white; 2=pale-pink; 3=deep pink; 4=carmine; 5=light purple; 6=deep purple |
| Color of upper corolla | CUP | At flowering | visual examination (scoring) 1=white; 2=pale-pink; 3=deep pink; 4=carmine; 5=light purple; 6=deep purple |
| Color of lower corolla | CLC | At flowering | visual examination (scoring) 1=white; 2=pale-pink; 3=deep pink; 4=carmine; 5=light purple; 6=deep purple; 7=deep carmine |
| Basal corolla ring diameter (mm) | BCRD | At flowering | <2 = narrow; 2-5=medium; >5=broad; Measured using ImageJ software |
| Pistil length (mm) | PIL | At flowering | <2=narrow; 3-6=medium; >6=long; Measured using ImageJ software |
| Stamen length (mm) | SL | At flowering | <2=narrow; 2-5=medium; >5=broad; Measured using ImageJ software |
| Flower numbers | FN | After flowering for 5 months | visual examination (scoring) <5=few; 5-15=medium; >15=high |
| Pedicel length (cm) | PEL | At flowering | <5 = short; 5-10=medium; >10=long; Measured using ImageJ software |
| Pedicel coiling | PEC | Fruit formation | 0= absent; 1=present |
| Darker petal margin | DPM | At flowering | visual examination (scoring) 1=present; 0=absent |
| Stigma position | SP | At flowering | visual examination (scoring) 0=non-exerted; 1=exerted |
| Leaf number | LN | 6 months after emergence | Number of leaves per plant was counted and recorded |
| Lamina length (cm) | LL | At leaf opening | <3=Short; 3-6= medium; >6=long; Measured using ImageJ software |
| Lamina width (cm) | LW | At leaf opening | <3=Short; 3-6= medium; >6=long; Measured using ImageJ software |
| Leaf length/width ratio | LL/W ratio | At leaf opening | LL/LW ratio = LL/LW |
| Leaf shape | LS | At leaf opening | visual examination (scoring) 1=orbicular (round, ovate); 2=cordate (heart-shaped); 3=reniform (kidney-shaped); 4=triangular |
| Leaf margin | LM | At leaf opening | visual examination (scoring) 1=entire (even, smooth throughout); 2=dentate (with symmetrical teeth); 3=scalloped (like toothed) |
| Lamina (degree of lobing) | LD | At full leaf opening | visual examination (scoring) 1=weak; 2=medium; 3=strong |
| Leaf pattern | LP | At full leaf opening | visual examination (scoring) 0=absent; 1=present |
| Leaf pattern collor | LPC | At full leaf opening | visual examination (scoring) 1=light green; 2=silver; 3=yellow-green; 4=green; 5=silver-green; 6=purple/pink |
| Petiole length | PETL | At full leaf opening | <5=Short; 5-10= medium; >10=long; Measured using ImageJ software |
| Leaf abaxial surface color | LASC | At full leaf opening | visual examination (scoring) 1=light green; 2=green:pink; 3=purple:pink; 4=green; 5=red/purple; 6=dark purple; |
| Leaf pattern area (cm) | LPA | At full leaf opening | <3=small; 3-5=medium; >5=large; Measured using ImageJ software |
| Leaf area | LA | At full leaf opening | <5=small; 5-10=medium; >10=large |
| Canopy architecture | CAR | 2 months after emergence | predominant structural arrangement of petioles and leaf distribution giving a specific shape to the above ground part of the plant was assessed visually as: 1=<5 petioles and low canopy architecture; 2=5-15 petioles and medium canopy architecture; 3=>15 petioles and strong canopy architecture |
| Canopy area | CA | In full bloom | Measured using ImageJ software |

**Supplementary Table S3.** Eigenvalues, variance, cumulative variance, and principal component scores (Eigenvectors) of the first ten components of phenotypic variation.

| Variables | PC 1 | PC 2 | PC 3 | PC 4 | PC 5 | PC 6 | PC 7 | PC 8 | PC 9 | PC 10 |
| --- | --- | --- | --- | --- | --- | --- | --- | --- | --- | --- |
| NS | **0.738** | 0.002 | -0.432 | 0.219 | 0.030 | 0.034 | 0.173 | 0.069 | -0.010 | 0.171 |
| PD | 0.255 | 0.128 | **0.464** | -0.150 | -0.096 | -0.291 | 0.292 | -0.348 | 0.428 | 0.316 |
| PETC | 0.347 | 0.315 | -0.382 | 0.232 | -0.141 | -0.121 | -0.290 | -0.010 | 0.191 | 0.220 |
| PV | 0.403 | 0.217 | -0.200 | 0.270 | 0.061 | **0.549** | -0.140 | -0.257 | -0.204 | 0.204 |
| FI | **0.737** | 0.174 | -0.340 | 0.199 | 0.304 | 0.029 | -0.045 | -0.013 | 0.172 | -0.003 |
| PL | **0.636** | -0.166 | 0.034 | -0.325 | -0.273 | 0.205 | 0.305 | 0.058 | -0.209 | 0.226 |
| PW | 0.408 | 0.384 | 0.107 | -0.440 | -0.051 | **0.484** | -0.110 | 0.012 | -0.080 | -0.001 |
| FA | **0.728** | 0.013 | 0.255 | -0.340 | -0.261 | 0.105 | -0.081 | 0.008 | 0.138 | -0.071 |
| PCF | 0.084 | **0.862** | 0.153 | 0.076 | 0.034 | -0.211 | -0.041 | 0.093 | -0.008 | 0.043 |
| PCS | 0.132 | **0.786** | 0.357 | 0.141 | 0.091 | -0.236 | -0.025 | 0.001 | -0.105 | 0.204 |
| BCRC | 0.074 | **0.651** | 0.144 | -0.299 | 0.327 | -0.247 | -0.027 | 0.385 | -0.032 | 0.051 |
| CUP | 0.210 | **0.752** | 0.206 | 0.081 | -0.142 | 0.158 | 0.111 | 0.231 | -0.004 | 0.158 |
| CLC | -0.255 | **0.803** | 0.146 | 0.008 | -0.219 | 0.125 | 0.219 | 0.108 | -0.066 | 0.066 |
| BCRD | **0.648** | 0.317 | -0.017 | -0.357 | -0.171 | 0.178 | 0.091 | -0.043 | -0.099 | -0.316 |
| PIL | 0.168 | 0.193 | 0.028 | -0.496 | 0.179 | 0.060 | -**0.516** | -0.417 | -0.271 | 0.047 |
| SL | **0.556** | 0.131 | 0.201 | -0.476 | -0.075 | -0.247 | 0.308 | -0.184 | 0.107 | -0.257 |
| FN | **0.446** | -0.202 | -0.637 | 0.286 | 0.273 | 0.003 | 0.258 | 0.027 | 0.129 | 0.132 |
| PEL | **0.543** | -0.393 | 0.058 | -0.086 | -0.459 | -0.356 | 0.093 | 0.144 | -0.023 | 0.169 |
| PEC | -**0.797** | -0.056 | 0.279 | 0.119 | -0.151 | -0.045 | 0.114 | -0.238 | 0.064 | 0.136 |
| DPM | 0.002 | 0.161 | 0.160 | 0.395 | **0.664** | -0.205 | -0.079 | 0.333 | -0.108 | -0.061 |
| SP | 0.434 | 0.094 | -0.100 | **-0.516** | 0.272 | 0.066 | 0.086 | -0.001 | 0.292 | 0.059 |
| LN | **0.797** | 0.089 | -0.381 | 0.237 | 0.080 | 0.049 | 0.054 | -0.015 | -0.007 | 0.101 |
| LL | 0.423 | -0.298 | **0.601** | 0.392 | -0.012 | 0.247 | -0.234 | 0.083 | -0.113 | 0.133 |
| LW | 0.439 | -0.249 | 0.451 | **0.544** | -0.258 | 0.281 | -0.121 | 0.201 | -0.022 | -0.075 |
| LL/Wratio | 0.018 | 0.221 | -0.267 | 0.305 | -0.435 | 0.159 | 0.354 | 0.260 | 0.190 | **-0.481** |
| LS | 0.185 | -0.207 | -0.067 | -0.397 | 0.098 | 0.189 | -0.340 | **0.431** | 0.424 | 0.077 |
| LM | **0.517** | -0.089 | 0.085 | 0.351 | 0.091 | -0.453 | 0.093 | -0.166 | -0.138 | -0.150 |
| LD | **0.587** | -0.140 | 0.199 | 0.139 | -0.007 | -0.303 | -0.432 | -0.009 | 0.113 | -0.177 |
| LP | 0.002 | -0.291 | 0.316 | -0.370 | **0.530** | 0.109 | 0.360 | 0.161 | -0.138 | -0.082 |
| LPC | -0.295 | **-0.465** | 0.114 | -0.150 | 0.302 | 0.303 | 0.370 | 0.121 | -0.088 | 0.239 |
| PETL | 0.046 | -0.328 | -0.199 | -0.217 | -0.404 | -0.359 | 0.038 | 0.304 | -0.246 | **0.428** |
| LASC | -**0.504** | 0.463 | 0.175 | 0.394 | -0.024 | 0.214 | 0.318 | -0.119 | 0.105 | 0.086 |
| LPA | 0.238 | -0.325 | **0.670** | 0.094 | 0.314 | 0.110 | 0.079 | 0.002 | 0.373 | 0.076 |
| LA | 0.596 | -0.193 | **0.613** | 0.379 | -0.140 | 0.099 | -0.037 | -0.038 | 0.038 | -0.003 |
| CAR | **0.639** | 0.038 | -0.142 | 0.199 | 0.289 | -0.011 | 0.335 | -0.321 | -0.042 | -0.010 |
| CA | **0.571** | -0.148 | 0.270 | -0.046 | 0.092 | -0.191 | 0.194 | 0.092 | -0.483 | -0.133 |
| Eigenvalue | 7.891 | 4.8176 | 3.5143 | 3.3535 | 2.3722 | 1.9996 | 1.9033 | 1.4036 | 1.3249 | 1.1939 |
| % variance | 21.7972 | 13.782 | 9.7621 | 9.3154 | 6.5896 | 5.5547 | 5.287 | 3.899 | 3.6804 | 3.3165 |
| Cumulative variance (%) | 21.7972 | 35.5792 | 45.341 | 54.656 | 61.246 | 66.801 | 72.088 | 75.987 | 79.667 | 82.984 |

**Supplementary Table S4.** Summary statistics of the 43 phenotypic traits of the 32 Cyclamen genotypes

| Variables | Mean | Std. error | Median | Min | Max | Kurtosis |
| --- | --- | --- | --- | --- | --- | --- |
| NS | 22.23031 | 2.13782 | 19 | 7 | 55 | 0.844125 |
| PD | 0.917734 | 0.680274 | 0.224 | 0.11 | 22 | 31.95849 |
| PETC | 4.09375 | 0.373949 | 3 | 2 | 8 | -0.86084 |
| PV | 2.34375 | 0.096401 | 2 | 1 | 3 | -0.76735 |
| FI | 4.21875 | 0.344116 | 3 | 2 | 9 | -0.49403 |
| PL | 5.279198 | 0.378063 | 5.15 | 2.2 | 10.12 | -0.89705 |
| PW | 3.963656 | 0.522032 | 3.35 | 0.45 | 13.5 | 2.513626 |
| FA | 9.678883 | 1.232746 | 6.2735 | 2.416 | 26.413 | 0.331893 |
| PCF | 3.3125 | 0.299319 | 3 | 1 | 6 | -1.06059 |
| PCS | 4.71875 | 0.416965 | 4 | 1 | 9 | -1.21255 |
| BCRC | 2.84375 | 0.304897 | 3 | 1 | 7 | -0.5631 |
| CUP | 2.09375 | 0.175973 | 2 | 1 | 5 | 0.843256 |
| CLC | 3.125 | 0.283092 | 3 | 1 | 7 | -0.01867 |
| BCRD | 0.865422 | 0.054505 | 0.85 | 0.45 | 1.515 | -1.00551 |
| PIL | 0.776875 | 0.364247 | 0.404 | 0.11 | 12.006 | 31.22598 |
| SL | 0.533 | 0.048871 | 0.4365 | 0.21 | 1.259 | 0.329899 |
| FN | 5.194604 | 0.576115 | 4.5 | 0.394 | 15 | 1.812741 |
| PEL | 11.78491 | 0.807223 | 10.76933 | 4.81 | 26.02667 | 1.873671 |
| PEC | 0.5625 | 0.089098 | 1 | 0 | 1 | -2.06327 |
| DPM | 0.1875 | 0.070102 | 0 | 0 | 1 | 0.8771 |
| SP | 0.5 | 0.089803 | 0.5 | 0 | 1 | -2.13793 |
| LN | 16.96875 | 1.490213 | 17 | 5 | 38 | -0.14032 |
| LL | 3.672603 | 0.327966 | 3.215875 | 1.367 | 11.82 | 11.94748 |
| LW | 3.574768 | 0.301451 | 3.038333 | 1.265 | 9.8875 | 6.1611 |
| LL/W ratio | 1.001663 | 0.039922 | 0.975636 | 0.365607 | 1.431443 | 0.933574 |
| LS | 2.03125 | 0.164883 | 2 | 1 | 4 | -0.16918 |
| LM | 2.1875 | 0.137939 | 2 | 1 | 3 | -1.2437 |
| LD | 1.53125 | 0.148815 | 1 | 0 | 3 | -0.50922 |
| LP | 0.84375 | 0.065213 | 1 | 0 | 1 | 2.077752 |
| LPC | 2.25 | 0.283981 | 2 | 0 | 6 | 0.016101 |
| PETL | 8.661107 | 0.664548 | 7.691333 | 4.5 | 18.95 | 1.164461 |
| LASC | 3.1875 | 0.247722 | 3 | 1 | 6 | -0.75545 |
| LPA | 3.985482 | 0.797373 | 2.7746 | 0 | 17.54333 | 2.160137 |
| LA | 13.60053 | 2.297656 | 8.141708 | 1.766667 | 63.4775 | 6.460964 |
| CAR | 1.90625 | 0.137367 | 2 | 1 | 3 | -1.28745 |
| CA | 152.0834 | 14.36983 | 146.1103 | 22.91 | 326.103 | -0.68151 |

**Supplementary Table S5.** Summary statistics of the color measurements in the flowers of 32 Cyclamen genotypes

| Measured part | CIELab Coordinate | Mean | Std. error | Median | Min | Max | Kurtosis |
| --- | --- | --- | --- | --- | --- | --- | --- |
| Slip | L* | 68.59375 | 3.090989 | 72.15 | 23.6 | 99.7 | 0.723989 |
|  | a* | 27.53438 | 4.098359 | 24.25 | -1.9 | 72.8 | -1.02273 |
|  | b* | -9.45 | 2.535013 | -10.75 | -39.3 | 21.9 | -0.18981 |
|  | C* | 32.24375 | 4.128303 | 28 | 0 | 74.2 | -1.08897 |
|  | h | 255 | 22.16957 | 326.5 | 3 | 347 | -0.58799 |
| Eye | L* | 46.70313 | 3.64739 | 43.55 | 10 | 85.2 | -0.74605 |
|  | a* | 36.14687 | 2.948985 | 38.75 | 0 | 69.4 | 0.014852 |
|  | b* | -8.13438 | 2.042535 | -9.15 | -29.3 | 16.5 | -0.17086 |
|  | C* | 38.80312 | 2.930518 | 41.45 | 1.1 | 71.4 | -0.13304 |
|  | h | 300.75 | 18.69303 | 339.5 | 8 | 359 | 3.978572 |
| Sepal | L* | 44.82813 | 2.284195 | 44.2 | 17.9 | 76.4 | -0.14075 |
|  | a* | 6.9375 | 2.054164 | 4.7 | -8.6 | 38.6 | 0.335492 |
|  | b* | 11.575 | 1.805525 | 8.4 | -4 | 43.8 | 1.698609 |
|  | C* | 18.1 | 1.791602 | 16.45 | 2.5 | 44 | 0.202648 |
|  | h | 71.09375 | 11.2451 | 70 | 5 | 154 | 12.31526 |
| Petiole | L* | 41.89687 | 2.474542 | 39.6 | 19.9 | 68.6 | -0.75225 |
|  | a* | 10.65625 | 1.841359 | 10.4 | -5.9 | 32.3 | -0.59482 |
|  | b* | 13.62813 | 1.509508 | 13.95 | -1.5 | 33.6 | -0.42282 |
|  | C* | 20.19688 | 1.522383 | 19.8 | 3.7 | 42.8 | 0.604092 |
|  | h | 73.25 | 13.88148 | 50.5 | 9 | 354 | 9.287154 |

**Supplementary Table S6.** Summary statistics of the color measurements in the leaves of 32 Cyclamen genotypes

| Measured part | CIELab Coordinate | Mean | Std. error | Median | Min | Max | Kurtosis |
| --- | --- | --- | --- | --- | --- | --- | --- |
| Dark pattern | L* | 48.9875 | 2.380968 | 47.2 | 22.1 | 78.4 | -0.37056 |
|  | a* | -14.3938 | 1.957028 | -15.7 | -32.2 | 33.8 | 11.28049 |
|  | b* | 17.01875 | 1.879685 | 17.75 | -8.2 | 38.2 | 0.264411 |
|  | C* | 24.8125 | 1.875224 | 23.6 | 7.4 | 48.9 | -0.02585 |
|  | h | 141.3438 | 7.157306 | 136 | 96 | 246 | 22.36895 |
| Silvery pattern | L* | 33.76875 | 2.702629 | 31.05 | 12.9 | 87.6 | 3.571194 |
|  | a* | -15.3344 | 1.616508 | -17.4 | -27.3 | 20.7 | 6.768256 |
|  | b* | 17.46875 | 1.53656 | 18.8 | -0.4 | 28.4 | -0.7513 |
|  | C* | 24.52813 | 1.724797 | 27.5 | 6.4 | 38.8 | -0.97818 |
|  | h | 133.3031 | 4.629863 | 133.5 | 17.7 | 183 | 12.46726 |
| Petiole | L* | 44.92813 | 2.676869 | 43.2 | 13.7 | 73.5 | -0.80575 |
|  | a* | 4.165625 | 1.969099 | 5.9 | -16 | 23.4 | -1.06445 |
|  | b* | 20.63438 | 1.992782 | 18.8 | 4 | 42.6 | -0.73773 |
|  | C* | 23.9875 | 1.821897 | 22.25 | 7.4 | 43.6 | -0.75046 |
|  | h | 74.59375 | 5.694837 | 75 | 10 | 124 | -1.00717 |


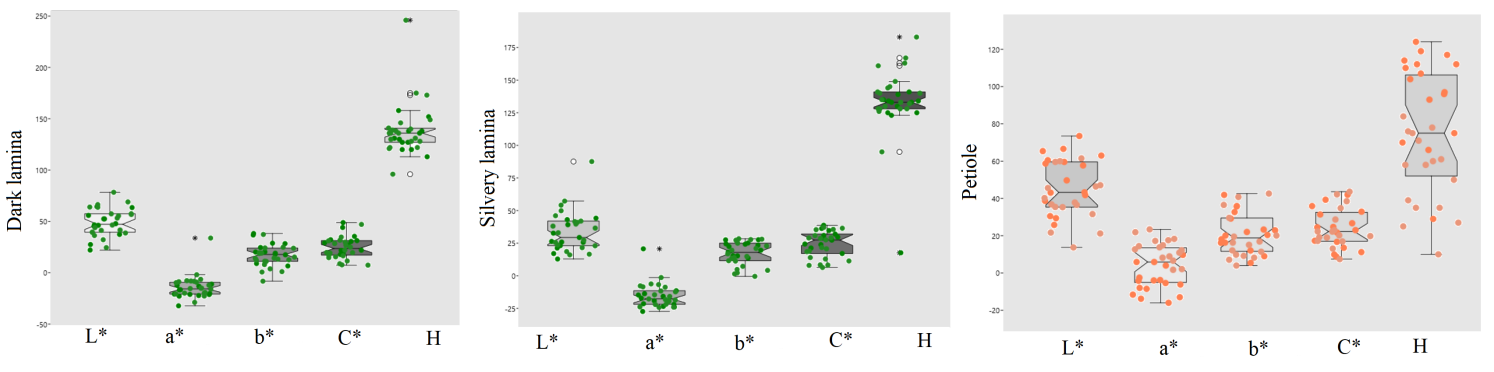


**Supplementary Figure 1**. Box plots of color characteristics of Cyclamen genotypes leaves


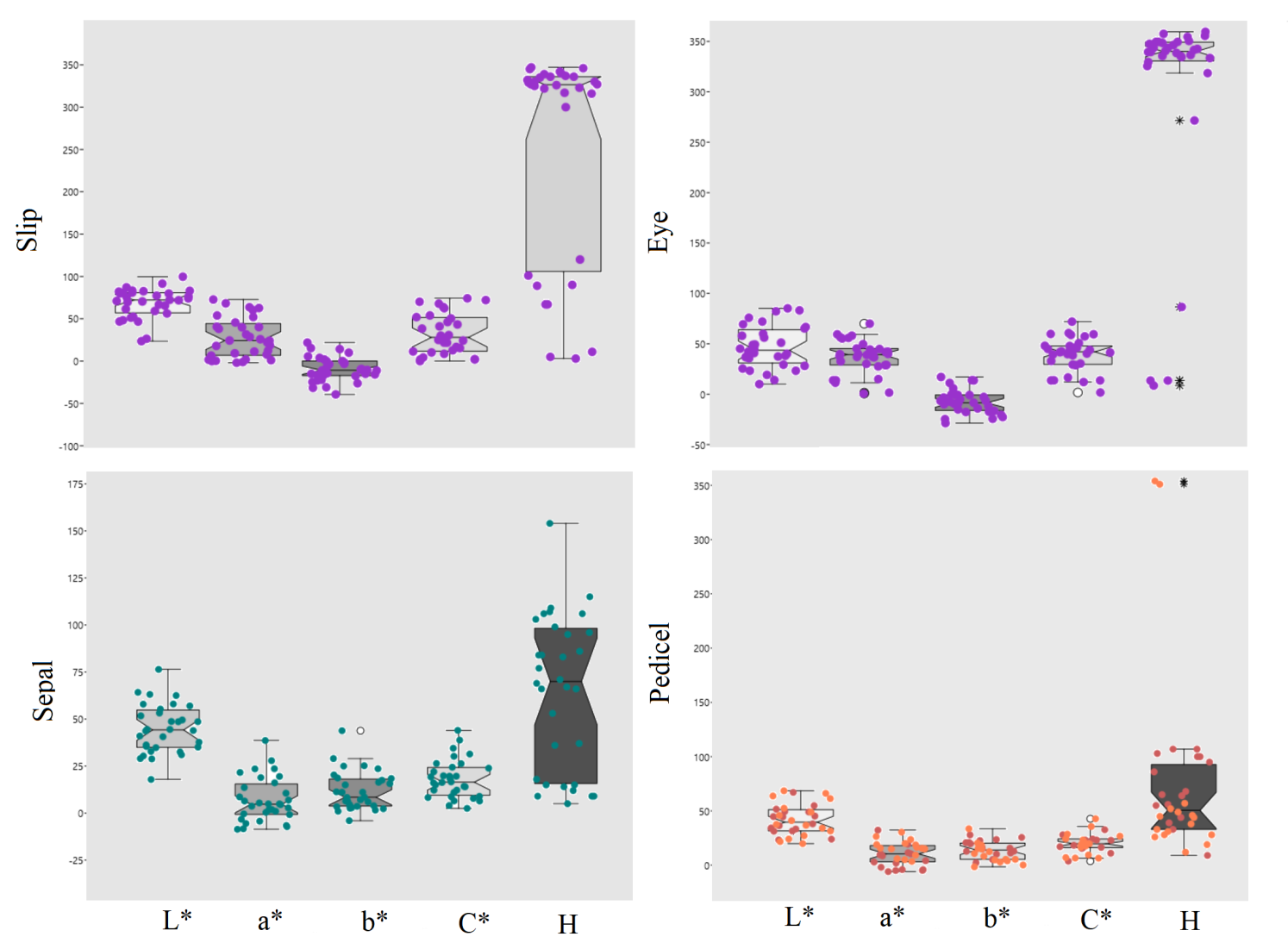


**Supplementary Figure 2**. Box plots of color characteristics of Cyclamen genotypes flowers

**Supplementary Table S7**. Description of the SRAP primer combinations selected for the 32 Cyclamen genotypes

| SRAP primer combination | Range | No. AB | No. PB | Polymorphism (%) | H | PIC | E | MI | D | R |
| --- | --- | --- | --- | --- | --- | --- | --- | --- | --- | --- |
| me1-em1 | 800-50 | 11 | 9 | 81.81 | 0.402 | 0.321 | 4.187 | 2.889 | 0.922 | 5.125 |
| me1-em2 | 1100-50 | 15 | 12 | 73.33 | 0.464 | 0.610 | 1.468 | 7.32 | 0.896 | 2.187 |
| me1-em3 | 1100-100 | 8 | 5 | 62.50 | 0.128 | 0.119 | 1.031 | 0.595 | 0.995 | 2.062 |
| me1-em4 | 1000-50 | 14 | 10 | 71.43 | 0.339 | 0.281 | 3.468 | 2.81 | 0.953 | 5.437 |
| me1-em6 | 1100-50 | 17 | 12 | 70.59 | 0.384 | 0.310 | 4.406 | 3.72 | 0.933 | 8.062 |
| me2-ba1 | 1300-50 | 17 | 13 | 76.47 | 0.309 | 0.261 | 3.062 | 3.393 | 0.963 | 5.978 |
| me2-em1 | 1200-100 | 13 | 11 | 84.61 | 0.283 | 0.243 | 2.906 | 2.673 | 0.971 | 5.062 |
| me2-em2 | 700-100 | 9 | 7 | 77.78 | 0.190 | 0.172 | 1.812 | 1.204 | 0.988 | 3.125 |
| me2-em3 | 1200-150 | 12 | 9 | 75.00 | 0.331 | 0.276 | 3.562 | 2.484 | 0.956 | 6.750 |
| me2-em4 | 1300-100 | 17 | 15 | 88.23 | 0.324 | 0.273 | 2.973 | 4.095 | 0.861 | 5.573 |
| me2-em6 | 900-50 | 12 | 9 | 75.00 | 0.187 | 0.170 | 1.781 | 1.53 | 0.989 | 3.562 |
| me3-em2 | 800-50 | 12 | 9 | 75.00 | 0.377 | 0.306 | 3.281 | 2.754 | 0.936 | 6.312 |
| me3-em6 | 900-50 | 15 | 11 | 73.33 | 0.368 | 0.300 | 3.402 | 3.3 | 0.941 | 6.187 |
| me4-ba1 | 800-50 | 13 | 10 | 76.92 | 0.327 | 0.274 | 3.718 | 2.74 | 0.957 | 6.818 |
| me4-em1 | 1500-100 | 17 | 13 | 76.47 | 0.241 | 0.212 | 2.531 | 2.756 | 0.980 | 5.062 |
| me4-em2 | 900-100 | 14 | 11 | 78.57 | 0.268 | 0.232 | 2.875 | 2.552 | 0.974 | 5.125 |
| me4-em3 | 900-50 | 16 | 9 | 56.25 | 0.335 | 0.279 | 3.843 | 2.511 | 0.954 | 6.937 |
| me4-em6 | 800-100 | 18 | 15 | 83.33 | 0.291 | 0.248 | 3.187 | 3.72 | 0.968 | 6.375 |
| me5-em1 | 1200-100 | 15 | 11 | 73.33 | 0.244 | 0.389 | 3.161 | 4.279 | 0.988 | 6.250 |
| me5-em2 | 900-50 | 15 | 10 | 66.66 | 0.236 | 0.208 | 2.468 | 2.08 | 0.981 | 4.312 |
| me5-em6 | 800-100 | 10 | 7 | 70.00 | 0.182 | 0.165 | 1.625 | 1.155 | 0.789 | 3.250 |
| me6-em1 | 1200-50 | 11 | 8 | 72.72 | 0.321 | 0.270 | 3.218 | 2.16 | 0.959 | 6.437 |
| me6-em2 | 900-100 | 9 | 6 | 66.66 | 0.235 | 0.207 | 2.312 | 1.242 | 0.681 | 4.625 |
| me6-em3 | 1000-50 | 12 | 10 | 83.33 | 0.314 | 0.264 | 3.125 | 2.64 | 0.962 | 6.250 |
| me6-em4 | 700-50 | 10 | 8 | 80.00 | 0.263 | 0.228 | 2.656 | 1.824 | 0.975 | 5.312 |
| me6-em6 | 1200-50 | 17 | 14 | 82.35 | 0.337 | 0.280 | 3.656 | 3.92 | 0.954 | 7.062 |
| Total |  | 349 | 264 |  |  |  |  |  |  |  |
| Mean |  | 13.4 | 10.1 | 75% | 0.30 | 0.27 | 2.91 | 2.78 | 0.93 | 5.35 |

Note: AB, amplified bands; PB, polymorphic bands; H, Genetic diversity; PIC, Polymorphism information content; E, effective multiple ratio; MI, maker index; R, resolving power.

**Supplementary Table** **S8**. Description of the SCoT primers selected for the 32 Cyclamen genotypes

| SCoT primer | Range | No. AB | No. PB | Polymorphism (%) | H | PIC | E | MI | D | R |
| --- | --- | --- | --- | --- | --- | --- | --- | --- | --- | --- |
| SCoT 1 | 2400-400 | 16 | 13 | 81.25 | 0.4028 | 0.3214 | 4.1875 | 4.1782 | 0.922 | 5.125 |
| SCoT 2 | 2000-200 | 20 | 17 | 80.00 | 0.2187 | 0.411 | 0.500 | 6.987 | 0.9852 | 1.324 |
| SCoT 3 | 2600-200 | 21 | 15 | 71.42 | 0.4967 | 0.3352 | 3.7812 | 5.028 | 0.7093 | 4.9375 |
| SCoT 4 | 2600-200 | 19 | 15 | 78.94 | 0.4992 | 0.404 | 4.812 | 6.06 | 0.8975 | 7.134 |
| SCoT 5 | 2000-800 | 5 | 4 | 80.0 | 0.1733 | 0.1582 | 1.4375 | 0.6328 | 0.991 | 2.875 |
| SCoT 6 | 2600-400 | 20 | 18 | 90.00 | 0.4803 | 0.38654 | 5.8125 | 6.95772 | 0.988 | 5.0625 |
| SCoT 7 | 2000-200 | 19 | 16 | 84.21 | 0.2371 | 0.209 | 2.0625 | 3.344 | 0.98134 | 4.125 |
| SCoT 8 | 2800-800 | 7 | 6 | 85.71 | 0.31577 | 0.2672 | 3.1234 | 1.6032 | 0.8893 | 2.654 |
| SCoT 13 | 2200-600 | 21 | 19 | 90.47 | 0.4725 | 0.4096 | 1.9855 | 7.7824 | 0.9834 | 4.153 |
| SCoT 16 | 1600-200 | 14 | 12 | 85.70 | 0.3884 | 0.3429 | 3.6400 | 4.1148 | 0.9345 | 4.2974 |
| SCoT 30 | 2800-600 | 23 | 19 | 82.60 | 0.4358 | 0.3408 | 4.8125 | 6.4752 | 0.8975 | 6.8254 |
| SCoT 33 | 2200-600 | 19 | 15 | 78.94 | 0.43837 | 0.4064 | 2.1754 | 6.096 | 0.9789 | 3.687 |
| Total |  | 204 | 169 |  |  |  |  |  |  |  |
| Mean |  | 17.0 | 14.1 | 82.43 | 0.38 | 0.33 | 3.19 | 4.93 | 0.93 | 4.35 |

Note: AB, amplified bands; PB, polymorphic bands; H, Genetic diversity; PIC, Polymorphism information content; E, effective multiple ratio; MI, maker index; RP, resolving power.


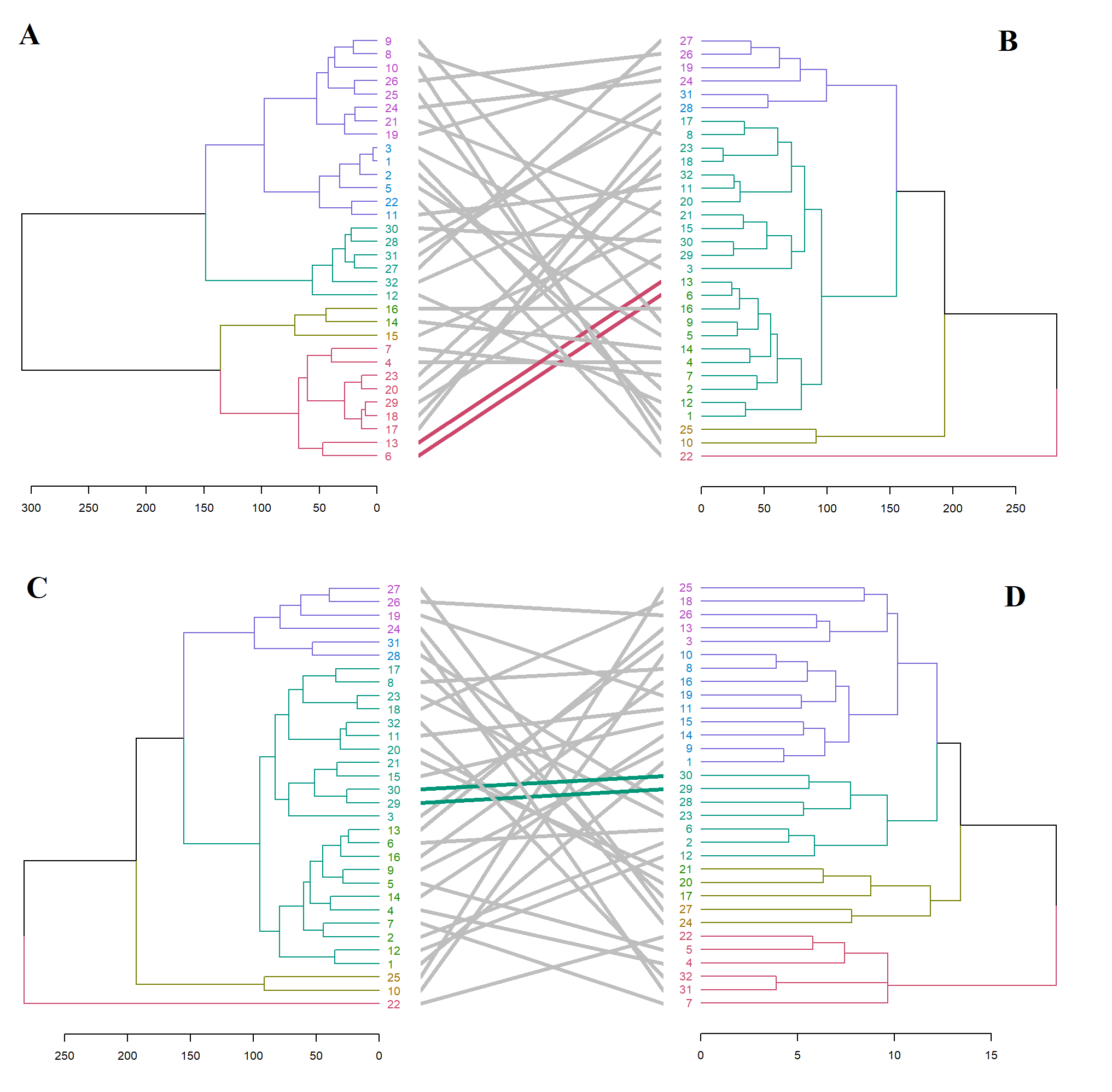


**Supplementary Figure 3**. Comparison of hierarchical clustering dendrograms of the 32 Cyclamen genotypes from phenotypic data (A) and color characters of leaves (B) with entanglement=0.59; color characters of leaves (C) and SCoT markers (D) with entanglement=0.47. The grey lines in between the dendrograms represent mismatched genotypes while the colored lines are genotypes that maintained the same position between clusters.

**
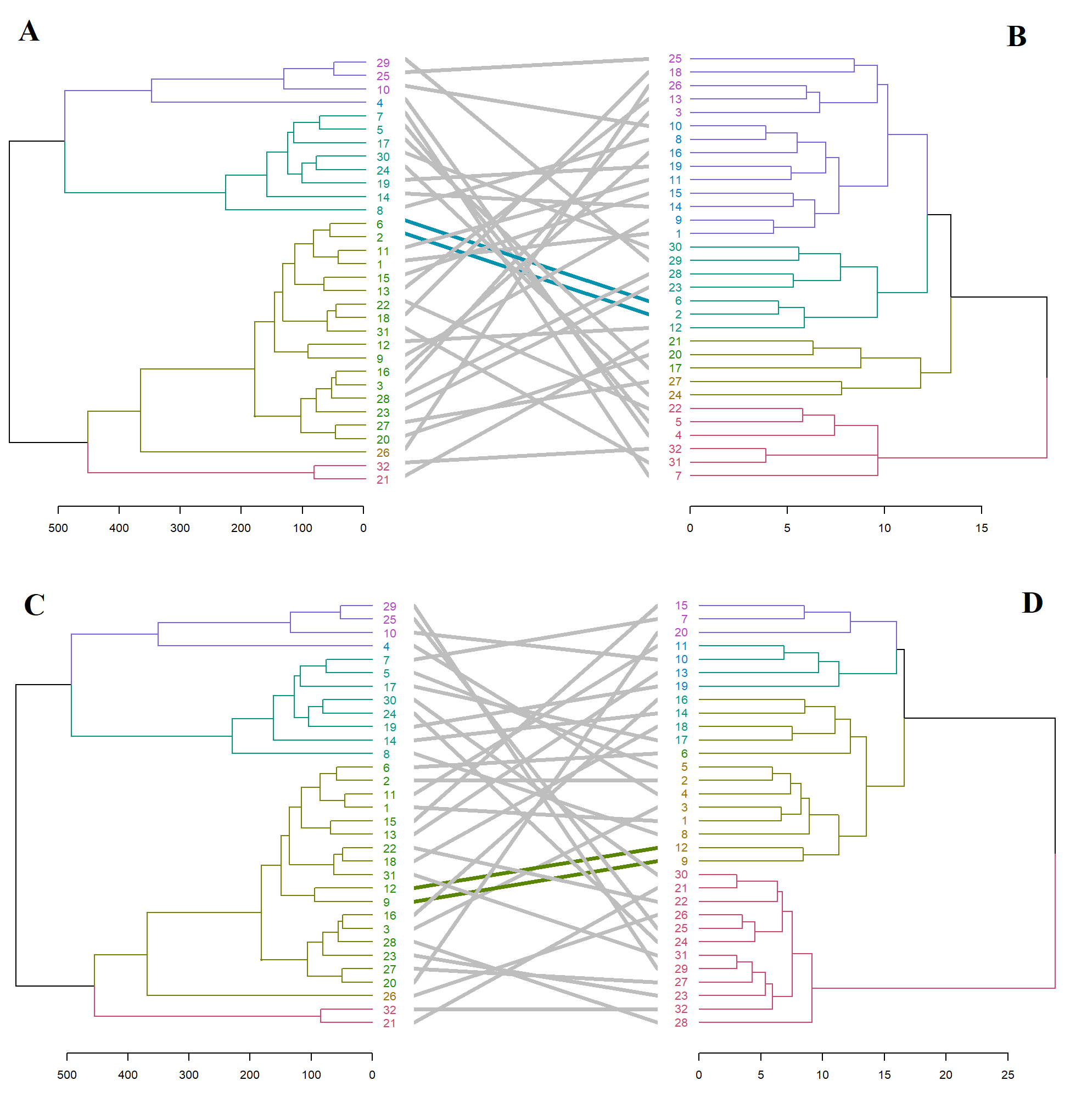
**

**Supplementary Figure 4**. Comparison of hierarchical clustering dendrograms of the 32 Cyclamen genotypes from color characters of flowers (A) and SCoT markers (B) with entanglement=0.45; and color characters of flowers (C) and SRAP markers (D) with entanglement=0.41. The grey lines in between the dendrograms represent mismatched genotypes while the colored lines are genotypes that maintained the same position between clusters.
